# *Mycobacterium tuberculosis* and *Paracoccidioides brasiliensis* Formation and Treatment of Mixed Biofilm *In Vitro*

**DOI:** 10.3389/fcimb.2021.681131

**Published:** 2021-11-01

**Authors:** Kaila Petronila Medina-Alarcón, Iara Pengo Tobias da Silva, Giovana Garcia Ferin, Marcelo A. Pereira-da-Silva, Caroline Maria Marcos, Mariana Bastos dos Santos, Luis Octávio Regasini, Marlus Chorilli, Maria José S. Mendes-Giannini, Fernando Rogerio Pavan, Ana Marisa Fusco-Almeida

**Affiliations:** ^1^ School of Pharmaceutical Sciences, Department of Clinical Analysis, Universidade Estadual Paulista (UNESP), Araraquara, Brazil; ^2^ Institute of Physics of Sao Carlos (IFSC)-University of Sao Paulo (USP) IFSC/USP, Sao Carlos, Brazil; ^3^ Exact Sciences and Engineering, Paulista Central University Center (UNICEP), Säo Carlos, Brazil; ^4^ Department of Chemistry and Environmental Sciences, Institute of Biosciences, Humanities and Exact Sciences, Universidade Estadual Paulista, São José do Rio Preto, Brazil; ^5^ Department of Drug and Medicines, School of Pharmaceutical Sciences, Universidade Estadual Paulista, Araraquara, Brazil; ^6^ Department of Biological, School of Pharmaceutical Sciences, Universidade Estadual Paulista, Araraquara, Brazil

**Keywords:** mixed biofilm, Mycobacterium tuberculosis, Paracoccidioides brasiliensis, nanoemulsion, 3’hydroxychalcone

## Abstract

Co-infection of *Mycobacterium tuberculosis* and *Paracoccidioides brasiliensis*, present in 20% in Latin America, is a public health problem due to a lack of adequate diagnosis. These microorganisms are capable of forming biofilms, mainly in immunocompromised patients, which can lead to death due to the lack of effective treatment for both diseases. The present research aims to show for the first time the formation of mixed biofilms of *M. tuberculosis* and *P. brasiliensis* (Pb18) *in vitro*, as well as to evaluate the action of 3’hydroxychalcone (3’chalc) -loaded nanoemulsion (NE) (NE3’chalc) against monospecies and mixed biofilms, the formation of mixed biofilms of *M. tuberculosis* H37Rv (ATCC 27294), 40Rv (clinical strains) and *P. brasiliensis* (Pb18) (ATCC 32069), and the first condition of formation (H37Rv +Pb18) and (40Rv + Pb18) and second condition of formation (Pb18 + H37Rv) with 45 days of total formation time under both conditions. The results of mixed biofilms (H37Rv + Pb18) and (40Rv + Pb18), showed an organized network of *M. tuberculosis* bacilli in which *P. brasiliensis* yeasts are connected with a highly extracellular polysaccharide matrix. The (Pb18 + H37Rv) showed a dense biofilm with an apparent predominance of *P. brasiliensis* and fragments of *M. tuberculosis*. PCR assays confirmed the presence of the microorganisms involved in this formation. The characterization of NE and NE3’chalc displayed sizes from 145.00 ± 1.05 and 151.25 ± 0.60, a polydispersity index (PDI) from 0.20± 0.01 to 0.16± 0.01, and zeta potential -58.20 ± 0.92 mV and -56.10 ± 0.71 mV, respectively. The atomic force microscopy (AFM) results showed lamellar structures characteristic of NE. The minimum inhibitory concentration (MIC) values of 3’hidroxychalcone (3’chalc) range from 0.97- 7.8 µg/mL and NE3’chalc from 0.24 - 3.9 µg/mL improved the antibacterial activity when compared with 3’chalc-free, no cytotoxicity. Antibiofilm assays proved the efficacy of 3’chalc-free incorporation in NE. These findings contribute to a greater understanding of the formation of *M. tuberculosis* and *P. brasiliensis* in the mixed biofilm. In addition, the findings present a new possible NE3’chalc treatment alternative for the mixed biofilms of these microorganisms, with a high degree of relevance due to the lack of other treatments for these comorbidities.

## 1 Introduction

Tuberculosis (TB) and paracoccidioidomycosis (PCM) are responsible for important systemic lung diseases and are distributed throughout the world and Latin America, respectively, especially in Brazil (World Health Organization, 2018). PCM is has a large presence in Latin America, most frequently in Argentina, Brazil, Colombia, Venezuela, Ecuador, and Paraguay, with an occurrence of 80% of cases in Brazil and a high incidence in the states of São Paulo, Paraná, Rio Grande do Sul, Goiás, and Rondônia (Martinez, 2017; Shikanai-Yasuda et al., 2017). Both diseases are important public health problems, with high mortality and morbidity indices (Ostrosky-Zeichner, 2012; Martín-Peña et al., 2014). *Mycobacterium tuberculosis* and *Paracoccidioides brasiliensis*, the etiological agents of these diseases, can be present as comorbidities, as has been documented in the literature, with an approximate occurrence of 2–20% (Shikanai-Yasuda et al., 2017). Currently, this infection is present in endemic and nonendemic areas due to migratory processes (Guery et al., 2015). In addition, this comorbidity can affect approximately 15–20% of immunocompromised patients (Quagliato et al., 2007; Martinez, 2017). There are few clinically reported cases of both diseases because the lack of medical records and similar symptoms can lead to misdiagnosis (Quagliato et al., 2007; Guery et al., 2015). Misdiagnosis can cause the death of the patient due to the lack of adequate treatment, which can be exacerbated by treatment resistance. This resistance can be caused by the formation of a mixed biofilm of these diseases (Sardi et al., 2014; Guery et al., 2015; Martinez, 2017). Thus, biofilms are organized and dynamic communities of microorganisms that strongly adhere to biological and non-biological surfaces, and serve as a niche for microorganisms and defense mechanisms against hostile environments, in addition to being reservoirs of persistent substances that can cause fatal infections (Costa-Orlandi et al., 2014; Del Pozo, 2018).

*Mycobacterium* spp. can form biofilms *in vitro* and *in vivo* with increased virulence and resistance to antibiotics, due to the difficulty of infusing the drug through the biofilm matrix. (Flores-Valdez et al., 2015; de la Cruz et al., 2020). According to Flores-Valdez et al., 2015, evaluations of chronic infections in rats have shown a tolerance to antibiotic drugs, which have been frequently related to the presence of biofilms in these infections (Flores-Valdez et al., 2015; Flores-Valdez, 2016).

According to Chakraborty et al. (2021), the formation of the *M. tuberculosis* biofilm was demonstrated in animal models in rats and primates as well as in sections of lung tissue obtained from patients with tuberculosis, demonstrating that the biofilm protects the bacilli from the immune system of the host, in addition to showing that isoniazid and rifampicin antimicrobial activity were reduced in infected mice, indicating the role of biofilms in phenotypic tolerance to drugs (Chakraborty et al., 2021).

The formation of the *M. tuberculosis* biofilm is facilitated by the aggregate-driven hydrophobicity which is associated with the presence of mycolic acids, GroEL1 chaperone, and glycopeptideglycans (Ojha et al., 2008; Nayak, 2015; Esteban and García-Coca, 2018).

Nayak et al. (2015) demonstrated the ability of *Mycobacterium avium* to form biofilms due to the presence of certain phospholipases (Nayak, 2015). Our research group demonstrated that *P. brasiliensis* is capable of forming biofilms *in vitro*; one of the factors that influence biofilm formation is the presence of hydrolytic enzymes and specific adhesins in the yeast biofilm (de Cássia Orlandi Sardi et al., 2015). Cattana and collaborators (2017), demonstrated the presence of a biofilm of*P. brasiliensis in vivo* in a 63-year-old patient with aorto-bifemural vascular prosthesis (Cattana et al., 2017).

Bacteria and *Candida albicans* are part of the intestinal microbiota, which can induce the formation of mixed biofilms in the blood circulation. According to a study conducted by Pornpimol et al. (2020), the formation of several monospecies biofilms such as *Acinetobacter baumannii* and *Pseudomonas aeruginosa* (PA) showed a formation less and more prominent, respectively. A mixed formation of *C. albicans* with *P. aeruginosa* (PA + CA) was shown to induce the formation of larger biofilms among the groups of mixed organisms and was even larger in the simultaneous formation of the same (Pornpimol et al., 2020).

Biofilm tolerance of antibiotics is multifactorial and is attributed to the low penetration of antibiotics, restricted growth with low oxygen tension, regulation of specific genes, and the presence of persistent cells (Ciofu et al., 2017). Thus, there is a need for the discovery of new compounds as chalcones, which are precursor substances for the synthesis of flavonoids and isoflavonoids with antitumor, antifungal, antibacterial, and anti-inflammatory properties (Pinho-Ribeiro et al., 2015; Wang et al., 2016).

Previous studies of our research group have demonstrated the potent antifungal activity of 2’hidroxychalcone against the fungus *P. brasiliensis* (Medina-Alarcón et al., 2020). According to Ramesh and collaborators, (2020) the indole chalcones presented antitubercular activity against the H37Rv strain of *M. tuberculosis* with MIC values of 210, 197, and 236 μM (Ramesh et al., 2020). The compound 3´hydroxychalcone (3’chalc) shows potent antifungal activity against *Cryptococcus gattii* in planktonic and biofilm forms *in vitro* and *in vivo* (Palanco et al., 2017). However, low solubility and instability allow chalcones to be incorporated into drug delivery systems to increase bioavailability, increase antimicrobial properties, and minimize the collateral effects (Talluri et al., 2016; Medina-Alarcón et al., 2017a; Yonashiro Marcelino et al., 2018). Among systems for chalcone release, nanoemulsions (NE) are widely used to improve their physicochemical properties (Čerpnjak et al., 2013; Kalepu et al., 2013; C. de Lima et al., 2020).

NE are heterogeneous systems that consist of an oil phase dispersed in an aqueous solution phase and stabilized by a surfactant or emulsifying agent; they have diameters that can vary from 50–200 nm. Those with smaller droplet sizes provide a large surface area to volume ratio, with faster absorption and greater uniformity in drug administration (Ganta et al., 2014; Medina-Alarcón et al., 2017b; dos Santos Ramos et al., 2019).

The NE of *Cymbopogon flexuosus* exhibited significant antimicrobial activity against *Mycobacterium fortuitum*, *Mycobacterium massiliense*, and *Mycobacterium abscessus* and was efficient against extant biofilm, while the free oil inhibited mycobacterial biofilm formation (Rossi et al., 2017).

A study was performed by Quatrin et al. on a NE containing *Eucalyptus gloubulus* oil, which did not present antimicrobial activity against *P. aeruginosa*. However, the NE was more efficient for *Candida* spp. biofilms than for oil-free (Quatrin et al., 2017).

It has been demonstrated that the microorganisms under study can form biofilms *in vitro* and *in vivo*; *P. brasiliensis* can form biofilms in patients, and *M. tuberculosis* can form biofilms in primates and lung tissue of patients, as has been reported previously. Although there are still no studies on the formation of mixed biofilms of these microorganisms, the comorbidity of PCM and tuberculosis is an alarming reality. Therefore, the present research aims to show for the first time the formation of the mixed biofilm of *M. tuberculosis* and *P. brasiliensis in vitro*, as well as to evaluate the action of NE loaded on 3’chalc (NE3’chalc) against monospecies and mixed biofilms.

## 2 Materials and Methods

### 2.1 Compounds

(E)-3´-hydroxychalcone was synthesized using Claisen Schmidt condensation with 3'-hydroxychalcone and benzaldehyde in a basic medium as outlined by Zeraik et al. (2012). The compound was identified using 1H-nuclear magnetic resonance and 13C (1H- and 13C-NMR), in which 1H-NMR displayed a coupling constant range (J) of H-α and H-β at 15.0 Hz, matching an (E)-diastereomer (Zeraik et al., 2012; Passalacqua et al., 2015; Palanco et al., 2017).

### 2.2 Strains and Growth Conditions

*M. tuberculosis* (Rv40) clinical strain, were taken from the Clemente Ferreira Institute National Center for Tuberculosis Treatment Reference, located in the city of São Paulo-SP-Brasil. The *M. tuberculosis* H37Rv (ATCC 27294) strain and *P. brasiliensis* strains of the phylogenetic species *P. brasiliensis* S1, isolate 18 (ATCC 32069). The strains used were from the collection of the Clinical Mycobacteriology and Mycology Laboratories of the School of Pharmaceutical Sciences, UNESP. *M. tuberculosis* was cultured on Middlebrook 7H9 broth (Difco) supplemented with 10% OADC enrichment (dextrose, albumin, and catalase — BBL/Becton-Dickinson), incubated for 10 days at 37°C at 5% CO_2,_ and the *P. brasiliensis* strain was incubated in Fava-Netto medium at 37°C for 4 days (Fava Netto, 1976; da Silva et al., 2017).

### 2.3 Monospecies Biofilm Formation Assay

**Figure 1:**
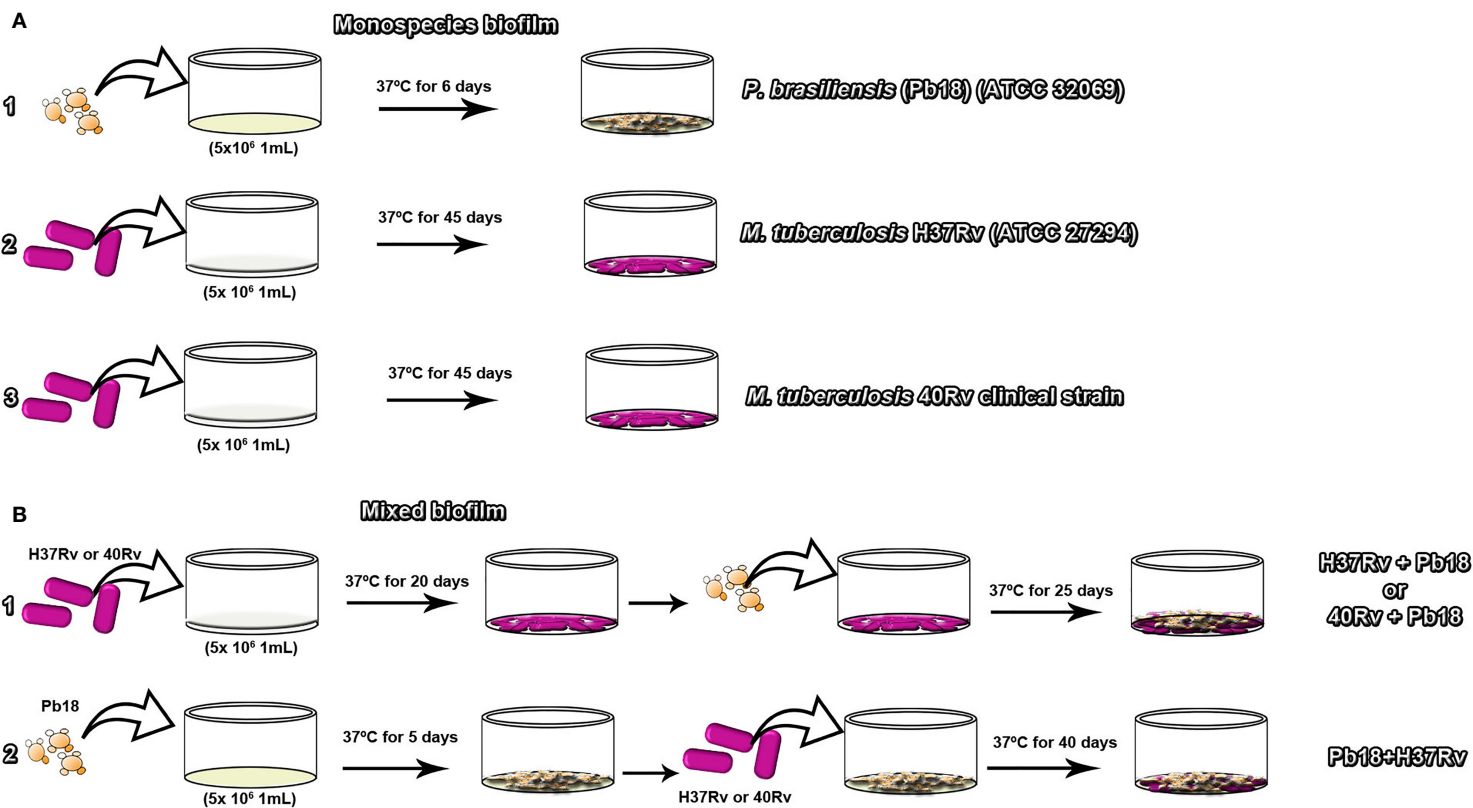
The experimental diagram of the formation of monospecies biofilms of *P. brasiliensis* (Pb18) in **(A.1)** *M. tuberculosis* H37Rv (ATCC 27294) in **(A.2)** and 40Rv clinical in **(A.3)** of mature biofilm formation respectively. Formation of mature mixed biofilms with 45 days of complete formation. The first condition of formation of *M. tuberculosis* H37Rv (ATCC 27294) and *P. brasiliensis* (Pb18) (H37Rv+Pb18) and *M. tuberculosis* 40Rv clinical strain and *P. brasiliensis* (Pb18) (40Rv + Pb18) in **(B.1)**. The second condition of formation of *P. brasiliensis* (Pb18) and *M. tuberculosis* H37Rv (Pb18 + H37Rv) in **(B.2)**.

Monospecies biofilms of *M. tuberculosis* H37Rv (ATCC 27294)(Rv40) clinical strain, and *P. brasiliensis* (Pb18) (ATCC 32069) were grown as previously described (Kulka et al., 2012; de Cássia Orlandi Sardi et al., 2015). The *M. tuberculosis* strains were grown on Middlebrook 7H9 broth (Difco) medium supplemented with OADC (BD/BBL ^®^) at pH 7.0, and *P. brasiliensis* inoculum was prepared on a modified fluid universal medium (FUM), as set to a final concentration of 5 × 106 CFU/mL for both microorganisms. Then, 1000 μL of inoculum was added to 24-well plates containing previously sterilized coverslips (13 mm in diameter) for microscopy evaluation, or 100 μL of inoculum was added to 96-well plates (TPP^®^, Trasadingen, Switzerland) for quantification evaluation of biofilms. The plate was incubated at 37°C for 45 days for *M. tuberculosis* and 6 days for *P. brasiliensis*. During this period, the culture medium of the wells was changed every 24 h. After this period, the supernatant was gently removed and washed 2–3 times with sterile saline to remove nonadherent cells. All assays were performed in triplicate in independent experiments in **Figure 1A** (1, 2, 3), respectively.

### 2.4 Mixed Biofilm Formation Assay

The mixed biofilm assay was performed according to the methodology described by Kulka et al. (2012) and Sardi et al. (2015) with some modifications. The strains were grown in Middlebrook 7H9 broth (Difco) supplemented with OADC (BD/BBL^®^) at pH 7.0. Two associations were performed; in the first association, the inoculum of *M. tuberculosis* H37Rv ATCC and 40 Rv clinic strain suspension were added independently to a 24-well plate (TPP^®^, Trasadingen, Switzerland) containing the previously sterilized coverslips for microscopic evaluation. For the kinetics evaluation, 96-well plates were used, to which 100 µL of inoculum was added. The plates were incubated without agitation at 37°C and 5% CO_2_ for 20 days. The supernatant was then gently removed and *P. brasiliensis* was added to each well containing the coverslips pre-covered with *M. tuberculosis* biofilms. The plates were incubated with the same parameters for another 25 days, forming mixed biofilms (H37Rv+Pb18) and (40Rv+Pb18), respectively in **Figure 1B.1**. In the second association, the inoculum of *P. brasiliensis* was initially prepared and incubated under the same conditions as the first association for five days. The supernatant was then gently removed, and *M. tuberculosis* was added to each well containing the coverslips pre-covered with *P. brasiliensis* biofilms and set to a final concentration of 5 × 10^6^ CFU/mL used for both microorganisms in the two association conditions. The plates were incubated with the same parameters for another 40 days, forming mixed biofilms (Pb18 + H37Rv) in **Figure 1B.2**. Both associations underwent biofilm formation for a total of 45 days. The supernatant was removed from the wells and washed 2–3 times with sterile saline to remove nonadherent cells. All assays were performed in triplicate in independent experiments.

### 2.5 Biofilms Quantification

#### 2.5.1 Determination of the Metabolic Activity Using XTT Reduction Assay

The XTT reduction assay (2.3-bis (2-methoxy-4-nitro-5-sulfophenyl)-5-[carbonyl (phenylamino)] 2H-tetrazolium hydroxide) measures the metabolic activity of the biofilm formation and treatment. For this assay, stock solutions of XTT (1 mg ml-1 on PBS) and menadione (1 mM on ethanol) were prepared. The biofilms were performed at different time points: the *M. tuberculosis* biofilm and mixed biofilm were evaluated every 5 days for 45 days, and the *P. brasiliensis* biofilms were evaluated every 24 h for 6 days. Then, 50 μL of XTT solution and 4 μL of a menadione solution were placed in each well of the 96-well plates, after which they were incubated at 37°C for 24 h for *M. tuberculosis* biofilms or 3 h for the *P. brasiliensis* biofilms. The metabolic activity of mitochondrial dehydrogenase reduces the tetrazolium salt XTT to a formazan salt, preventing a colorimetric change that is correlated with the cell viability of the microorganism. These colorimetric changes were measured using a Microplate Reader - Spectrophotometer (iMarkTM; BIO-RAD) at 490 nm (Pitangui et al., 2012; de Cássia Orlandi Sardi et al., 2015).

#### 2.5.2 Quantification of Biofilm Mass Using Crystal Violet Staining

Biofilm formation and treatment were quantified according to the technique described by (Mowat et al., 2007), with modifications. The biofilm assays were performed in 96-well plates (TPP^®^, Trasadingen, Switzerland). The biofilms were evaluated every 5 days for 45 days for the *M. tuberculosis* biofilm and mixed biofilm, and every 24 h for *P. brasiliensis* biofilms for 6 days. The culture medium was removed from each well, washed several times with PBS, and air-dried at room temperature. Then, 100 μL of 0.5% crystal violet solution was added to each well, and the plates were incubated for 20 min at room temperature. Unbound dye was removed by washing with sterile water to remove excess stain, the well was air-dried, and the residue from each well was dissolved in 100 μL of a 95% ethanol solution. This solution was homogenized and transferred to a new 96-well plate, which was read using a Microplate Reader - Spectrophotometer (Microplate Reader iMarkTM; BIO-RAD; Hercules, CA, USA) at a wavelength of 570 nm.

#### 2.5.3 Quantification of the Extracellular Matrix Using Safranin Staining

The extracellular matrix produced for monospecies and mixed biofilm formation and treatment was quantified using a safranin assay as previously reported (Seidler et al., 2008; Costa-Orlandi et al., 2014), with modifications. The biofilm was evaluated every 5 days for 45 days for the *M. tuberculosis* biofilm and mixed biofilm, and every 24 h for *P. brasiliensis* biofilms for 6 days. The biofilms were performed in 96-well plates (TPP^®^, Trasadingen, Switzerland). The culture medium was removed from each well, washed three times with PBS, and air-dried at room temperature. Subsequently, 100 μL of 1% safranin solution was added to each well and incubated for 20 min at room temperature. The plates were washed until the supernatant was clean. Finally, the plates were read on a Microplate Reader - Spectrophotometer (Microplate Reader iMarkTM; BIO-RAD, Hercules, CA, USA) at a wavelength of 490 nm.

### 2.6 Scanning Electron Microscopy

All biofilms formed on coverslips were performed as previously reported (Morris et al., 1997; Pitangui et al., 2012) with modifications. After the formation or treatment of biofilms, the plates were washed three times with PBS to remove the planktonic cells. The plates were fixed with 1000 μL of glutaraldehyde solution (Sigma-Aldrich, St Louis, MO, USA) at 2.0% to *P. brasiliensis* biofilm overnight and at 5.0% to *M. tuberculosis* biofilm and mixed biofilm for 3 days at 4°C. All biofilms were washed with PBS, followed by dehydration at concentrations of 50–100% ethanol at room temperature. The samples were dried with a Samdri 780A desiccator (Rockville, MD, USA). Subsequently, all samples were assembled in cylinders arranged in a high vacuum evaporator (Denton Vacuum Desk V, Jeol, Moorestown, NJ, USA) to form the gold coating. All topographic images of the biofilms were analyzed using a scanning electron microscope (Jeol JSM-6610LV, Peabody, MA, USA) at the Instituto de Química de Araraquara, UNESP.

### 2.7 Multiplex PCR for Detection of *Paracoccidioides brasiliensis* and *Mycobacterium tuberculosis* in Mixed Biofilm

In this study, genomic DNA from all samples was isolated according to the glass bead protocol described by Van Burik et al. (1998). This method was used for the detection and identification of *P. brasiliensis* and *M. tuberculosis* in polymicrobial biofilm samples. The PCR conditions used with selected primers (**Figure 6**) combined in the same reaction were optimized using touchdown-PCR with 50-µL reaction consisting of 10× PCR Buffer (GeneDireX^®^), 200 ng of DNA template, 0.2 µM of each primer, 200 µM of dNTP mix, 1.25 units of Taq DNA Polymerase (GeneDireX^®^), and DEPC-treated water. The touchdown PCR cycle conditions were as follows: 94°C for 5 min, 10 cycles of 30 s at 94°C, a temperature range of 65–55°C for 45 s, 30 cycles of 94°C for 30 s, 55 °C for 45 s, 2 min at 72°C and a final cycle at 72°C for 10 min. The reactions were carried out in a Veriti thermocycler (Carlsbad, CA, USA). The amplicons were analyzed using 0.8% agarose gel and visualized with GelRed Nucleic Acid Gel Stain (Uniscience) under UV light (Van Burik et al., 1998).

### 2.8 Incorporation of 3’hydroxychalcone in Nanoemulsion

The 3' synthesis of the NE and the incorporation of the 3';hydroxychalcone (NE3'chalc) was performed according to a previously published method (Medina-Alarcón et al., 2020 with minor modifications. The NE was composed of 8% oil phase (cholesterol), 10% surfactant (castor oil, polyoxyl60/PEG-hydrogenated, phosphatidylcholine [PS]), and sodium oleate at a proportion of 8:6:3. The aqueous phase was 80% phosphate solution (pH 7.4). The mixture was sonicated using a rod sonicator (Vibra Cell, Sonics and, Material Inc., CT, USA) at 500 W and 13 amplitude for 30 min with a discontinuous mode for 60 s at 30 s intervals in an ice bath throughout the sonication process. The NE was centrifuged at 11,180 × *g* for 15 min to eliminate the waste released due to the presence of the titanium rod sonicator. NE3'chalc at a final concentration of 2 mg/mL was prepared under the same conditions as NE. All formulations were sterilized with a membrane filter with a 0.22 µm pore size (Medina-Alarcón et al., 2020).

#### 2.8.1 Physical-Chemical Characterization of NE and NE3'Chalc

The droplet diameters, polydispersity index (PDI), and zeta potential were evaluated for NE and NE3'chalc. The samples were diluted (10 µL/mL) in deionized water. All samples were placed in an analysis chamber for laser analysis with a laser beam (λ = 633 nm) at a temperature of 25°C and an angle of 90°. The methodology was performed through the laser radiation dynamic spreading equipment – light scattering, using a Zetasizer Nano ZS particle analyzer (Malvern Instruments, Worcestershire, UK). The samples were evaluated three times, and the mean results were expressed as means and standard deviations (Jamil et al., 2016; Medina-Alarcón et al., 2020).

#### 2.8.2 Atomic Force Microscopy

A drop of NE and NE3'chalc was placed in a circular glass cover and allowed to dry at 25–30°C. The samples were tested with a rectangular silicone tip, a spring constant of 42 N/m, and a free oscillation of 330 kHz in intermittent contact. Atomic force microscopy (AFM-BRUKER Dimension ICON, Santa Barbara, CA, USA) was used for the analysis (Marquele-Oliveira et al., 2016).

### 2.9 Cytotoxicity Assay

Cytotoxicity assays of 3'chalc and NE3'chalc were evaluated in HepG2 (human liver carcinoma) cells, kindly provided by the Biotech Biotechnology Laboratory (UNICAMP, Sao Paulo Brazil). The cell lines were added to 96-well plates with each well containing 10^5^ cells. The plates were incubated at 36.5°C in 5% CO_2_ for 24 h to form a cell monolayer (Husøy et al., 1993). The formulations were analyzed at different concentrations ranging from 0.97–62.5 μg/mL in serial dilutions. Cytotoxicity was assessed using the 0.4% sulforhodamine B assay and measured using a microplate reader at 570 nm. A negative control with untreated cells was also used, and this reading was considered a baseline of 100% living cells. The results were evaluated using triplicates independent assays. The results were expressed using the formula below, with minor modifications (Rajpoot et al., 2012; Medina-Alarcón et al., 2017a).

Viable cells % = (test mean − white mean/mean negative control − mean white)×100

### 2.10 Anti-Mycobacterium Tuberculosis Assay

Anti-*Mycobacterium tuberculosis* activity was determined using the REMA methodology, as described by Palomino et al. (2002). The compounds were evaluated against *M. tuberculosis* H37Rv (ATCC 27294) and (Rv40) clinical strain, and diluted in Middlebrook 7H9 broth (Difco) supplemented with 10% OADC enrichment (dextrose, albumin, and catalase — BBL/Becton-Dickinson), with the final drug concentration ranging from 0.97–62.5 μgmL^-1^. Rifampin (Sigma-Aldrich^®^) was used as a standard drug. The inoculum was adjusted to 2 × 10^5^ CFU/mL^-1^, and 100 μL of the inoculum was added together with 100 μL of the formulations to each well of a 96-well microtiter plate. The plates were incubated for 7 days at 37°C. Then, 30 μL of 0.01% resazurin (Sigma-Aldrich^®^) was added to each well and incubated at 37°C for 24 h. The fluorescence was measured at wavelengths of 530 and 590 nm, for excitation and emission filters, respectively, and read in a cell imaging system, Cytation 3 (Biotek^®^). The assay was performed in triplicate in independent experiments (Palomino et al., 2002; da Silva et al., 2017).

#### 2.10.1 *In Vitro* Antifungal Activity

The minimum inhibitory concentration (MIC) was determined using the microdilution assay according to the standard reference method (CLSI) document M27-A3 and Medina-Alarcón et al. (2017a). The antifungal activity of formulations was evaluated against the isolates of the *P. brasiliensis*, at a final concentration of 1.5×10^3^ UFC/mL in RPMI-1640 medium (Sigma-Aldrich, MO, USA). The formulations were added in serial dilutions at concentrations from 0.24–250 μg/mL in a 96-well plate. The AMB (Sigma-Aldrich^®^) was used as drug control. The plates were incubated on a shaker at 37°C and 150 r.p.m. for 48 h. After this period, alamar blue (Sigma-Aldrich) was added to evaluate fungal cell viability. The plate was incubated for 24 h with a total of 72 h for the final. The fluorescence was measured at wavelengths of 530 and 590nm, with filters of excitations and emissions, respectively, and read in a cell imaging system, Cytation 3 (Biotek^®^) (Clinical and Laboratory Standards Institute, 2008; Medina-Alarcón et al., 2017a).

### 2.11 Effect of 3'chalc and NE3'chalc Against Monospecies and Mixed Biofilms

The biofilms were formed in 24-well plates for microscopic evaluation or in 96-well plates for colorimetric assay evaluation. The biofilms were incubated for 30 days with complete formation under the same conditions according to sections 2.3 and 2.4. Then the supernatant was removed and the plate was washed with sterile PBS, and the NE3'chalc and 3'chlac solutions were added at concentrations ranging from 0.25–64.0 µg/mL, placed in contact with the biofilms, and incubated together with their respective controls. The plates were incubated for 10 days at 37°C with 5% CO_2._ After this, the supernatant was removed again and washed with sterile PBS. The quantifications of the biomass and extracellular matrix of biofilms were performed using crystal violet and safranin staining respectively, and the metabolic activities (XTT reduction assay) were assessed. Results of at least 50% were compared to the biofilm growth control, which corresponded to 100% free-of-treatment. Finally, the biofilm treatment was evaluated using SEM. The assay was performed in triplicate in independent experiments.

### 2.12 Statistical Analysis

All assays were performed using GraphPad Prism 5.0 (CA, USA). The data analysis was performed using analysis of variance with Tukey post-test, with p-values of < 0.05, 0.01, and 0.001 considered statistically significant. The results are expressed as the mean ± standard deviation (SD).

## 3 Results

### 3.1 Biofilms Quantification

#### 3.1.1 Determination of the Metabolic Activity of the Biofilm Using the XTT Reduction Assay

**Figure 2:**
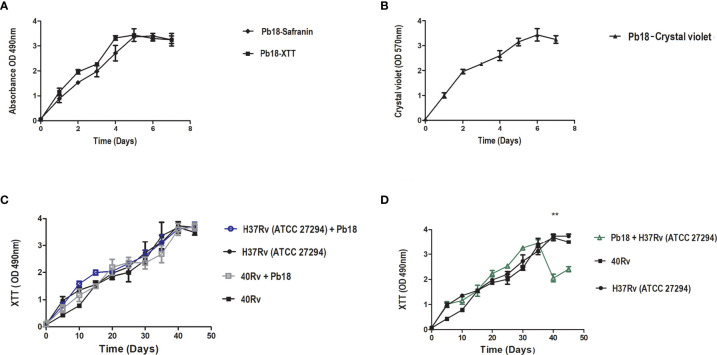
Kinetics formation evaluation of metabolic activity, quantification of biomass and extracellular matrix of *P. brasiliensis* (Pb18) biofilm metabolic activity and safranin staining in **(A)**. Quantification of biomass in **(B)**. Kinetics of formation of *M. tuberculosis* H37Rv (ATCC 27294) and 40Rv clinical strain of monospecies biofilm and mixed biofilms in the first formation condition (H37Rv + Pb18) and (40Rv + Pb18) and comparison between biofilms in **(C)** and the second condition of formation (Pb18 + H37Rv) and comparison with their monospecies biofilms in **(D)**. The experiments were performed independently. Mean ± SD was used in the data processing, and the differences between groups were analyzed for statistical significance used ANOVA, followed by Tukey’s test for comparisons between groups,** = p < 0.01 was considered significant.

To standardize the formation of biofilms, the metabolic activity of biofilms was evaluated using the XTT reduction assay method. **Figure 2A** shows the high metabolic activity of the monospecies biofilm, *P. brasiliensis*, with a significant increase in its activity after 2 days, tending to plateau after 4 days, and later showing a mature biofilm formation after 6 days. **Figure 2C** shows the biofilm of*M. tuberculosis* H37Rv and 40 Rv clinical strain, where the biofilms showed an increase in their metabolic activity within 20 days of formation, increasing steadily to reach a plateau within 40–45 days. No significant difference was observed (* = p < 0.05) between the two biofilms. In the mixed formation, the scheme of the first formation condition that was performed (the formation of (H37Rv + Pb18) and (40Rv + Pb18)] can be seen in **Figure 1B.1**. The results showed curves with a constant increase in metabolic activity throughout the formation, with an increase after 20 days when *P. brasiliensis* was added to the formation of the mixed biofilm. This also reached 45 days of formation with high production of metabolic activity, and its production was comparable to the monospecies biofilms, which did not show a significant difference (* = p < 0.05) as observed in **Figure 2C**. The second (Pb18 + H37Rv) mixed biofilm formation condition is shown in **Figure 1B.2**. This biofilm showed a constant increase in metabolic activity, reaching its maximum production in 30 days and with a decline after 35 days of formation, showing a significant difference only after 40 days of formation (* * = p <0.01) at this single point; however, it did not show a significant difference to the other formation points when compared to the monospecies biofilms (**Figure 2D**).

#### 3.1.2 Biomass and Extracellular Matrix Quantification Using Crystal Violet and Safranin Staining

Quantification of biomass and extracellular matrix of all biofilms was performed by staining with crystal violet and safranin. The quantification of the extracellular matrix of Pb18 biofilm observed in **Figure 2A** shows a significant increase after 2 days, with the complete formation of the mature biofilm within 6 days of formation, which is comparable to the results from crystal violet (**Figure 2B**), thus showing a similar behavior in these evaluations and corroborating the results of the evaluation of the metabolic activity during formation.

**Figure 3:**
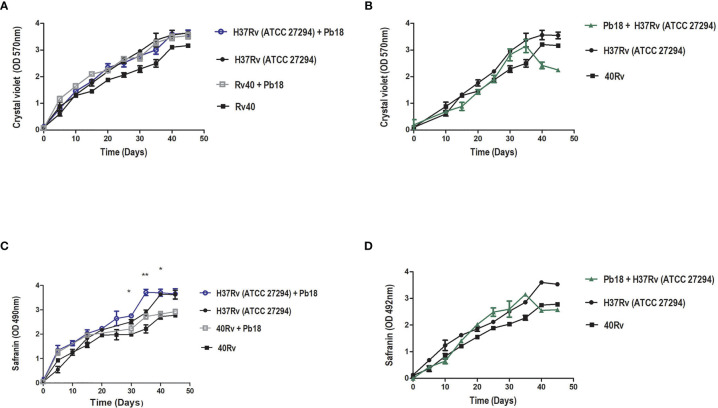
Biomass quantification using crystal violet staining and quantification of the extracellular matrix using safranin staining of *M. tuberculosis* H37Rv (ATCC 27294) and clinical strain (40Rv) monospecies biofilms and mixed biofilms in the first formation condition (H37Rv + Pb18) and (40Rv + Pb18) and comparison between the monospecies and mixed biofilms in **(A, C)**, respectively, and the second formation condition (Pb18 + H37Rv) and comparison with their monospecies biofilms in **(B)** and in **(D)**, respectively. The experiments were performed independently. Mean ± SD was used in the data processing, and the differences between groups were analyzed for significance using ANOVA, followed by Tukey’s test for comparisons between groups, (* = p < 0.05 and ** = p < 0.01) was considered significant.

Quantification of the biomass of *M. tuberculosis* H37Rv (ATCC 27294) and 40Rv clinical strains of monospecies biofilms and mixed biofilms (H37Rv +Pb18) and (40Rv +Pb18) in the first formation condition showed a high and constant production of biomass throughout the 45 days of training, reaching a plateau at 40 days. When comparing monospecies and mixed biofilms, there was no significant difference (* = p < 0.05) between these biofilms, as observed in **Figure 3A**. In the quantification of the biomass of the second condition of formation (Pb18 + H37Rv) of mixed biofilm, a constant increase in biomass was observed, reaching its maximum production after 30 days and with a decline after 35 days. When compared with the monospecies biofilms H37Rv and 40Rv, there was no significant difference (* = p < 0.05), as shown in **Figure 3B**. **Figure 3C** shows the quantification of the extracellular matrix of *M. tuberculosis* H37Rv (ATCC 27294) and the clinical strain 40Rv monospecies biofilms, as well as the formation of the first formation condition (H37Rv + Pb18) and (40Rv + Pb18) of mixed biofilms. This shows a high and constant production of extracellular matrix throughout the formation over 45 days, reaching a plateau at 35 days for the mixed biofilms and 40 days for the monospecies biofilms. When the mixed biofilms were compared with their respective monospecies biofilms, there was no significant difference (* = p < 0.05). However, when (H37Rv + Pb18) was compared with 40Rv, it showed significant difference (* = p < 0.05) at 30 and 35 days and (** = p < 0.01) at 40 days. The biofilms (H37Rv + Pb18) and (40Rv + Pb18) showed significant difference of (* = p < 0.05) at 30-40 days. These significant differences are shown exclusively in this assay. In the evaluation of the second condition of formation (Pb18 + H37Rv) of the mixed biofilm, a constant increase in the extracellular matrix was observed, reaching its maximum production at 30 days with a brief decline at 35 days but reaching a plateau after 40 days of formation, reaching complete biofilm formation after 45 days, which is the standard time used for all formations. When comparing the mixed biofilm and its monospecies biofilms, the highest extracellular matrix production was shown for the monospecies biofilm, with no significant difference (* = p < 0.05), as shown in **Figure 3D**.

### 3.2 SEM

**Figure 4:**
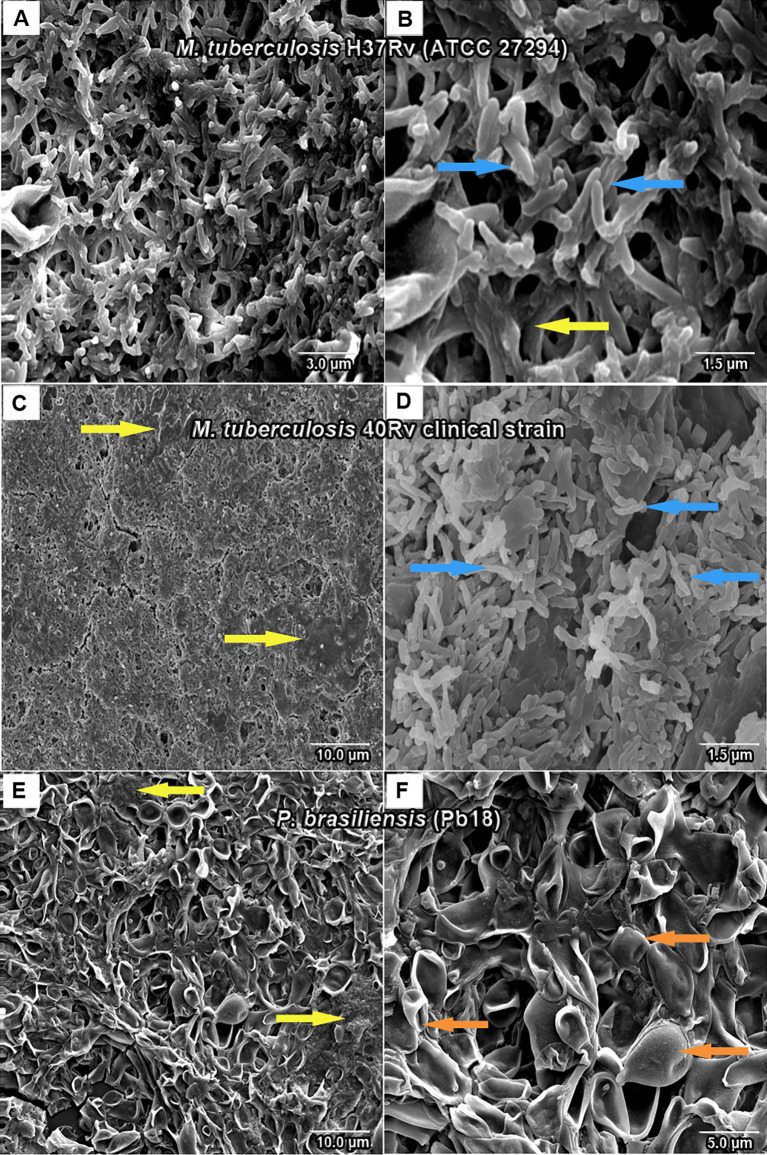
SEM images of mature monospecies of *M. tuberculosis* biofilm strain H37Rv (ATCC 27294) biofilm in **(A, B)**, the blue arrows denote *M. tuberculosis* bacillus and the extracellular matrix is shown in yellow arrows in **(B)**. Biofilm of the 40Rv clinical strain of *M. tuberculosis* in **(C, D)**, yellow arrows show the extracellular matrix in **(C)** and the blue arrows denote their bacilli in **(D)**. Biofilm of *P. brasiliensis* (Pb18) in **(E, F)**, the presence of extracellular matrix is shown in yellow arrows in **(E)** and yeast fungus is shown in orange arrows in **(F)**.

**Figure 5:**
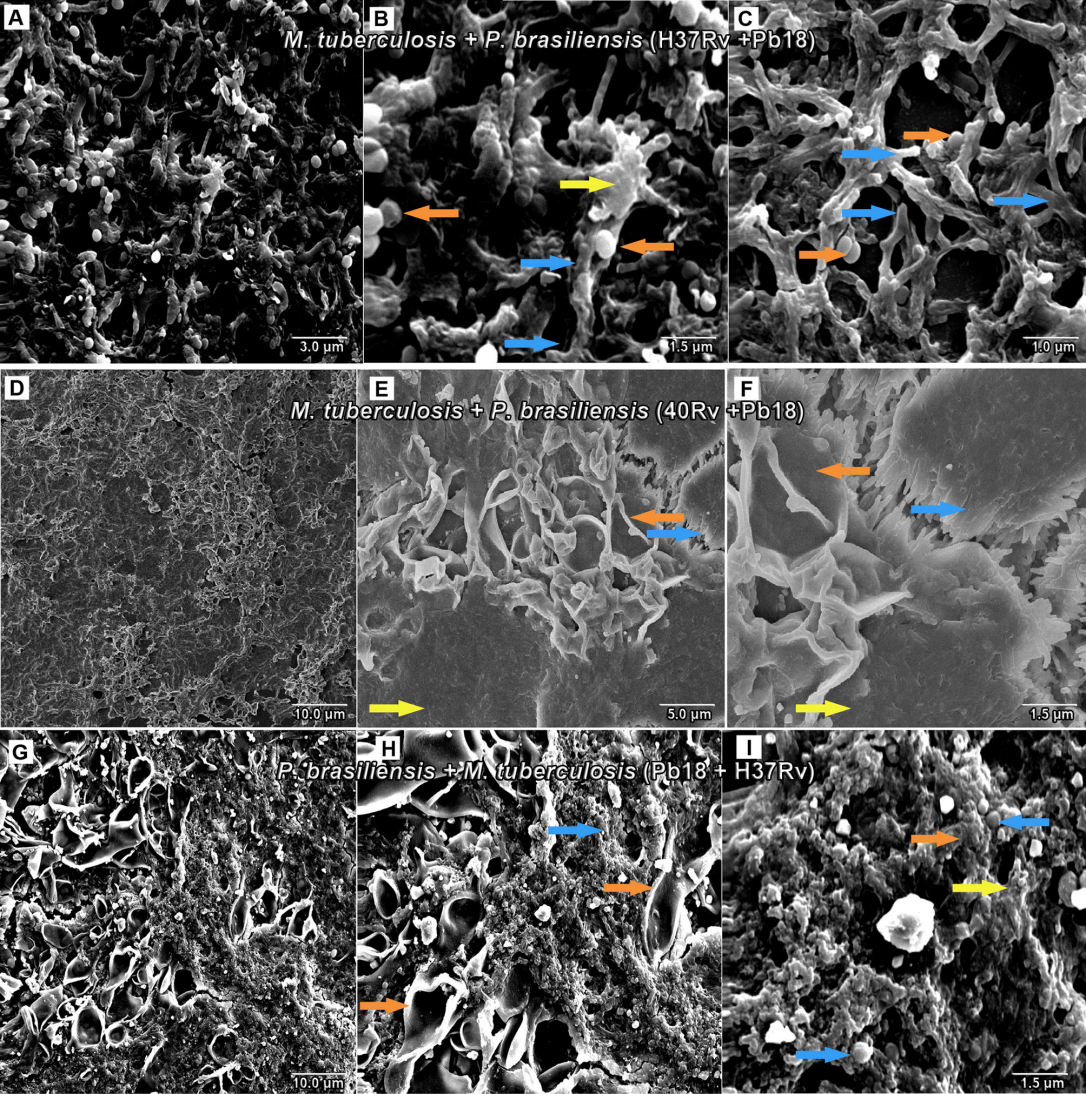
SEM images of mixed biofilms in the first condition of formation of *M. tuberculosis* H37Rv (ATCC 27294) and *P. brasiliensis* (Pb18) (H37Rv + Pb18) in **(A–C)**, the blue arrows show the H37Rv bacilli and the presence of yeasts (PB18) is shown in orange arrows in **(B, C)** and the presence of extracellular matrix is shown in yellow arrows in **(B)**. Clinical strain *M. tuberculosis* 40Rv and *P. brasiliensis* (Pb18) (40Rv + Pb18) biofilm in **(D–F)**, the blue arrows denote 40Rv bacilli and the orange arrows denote fungi from (Pb18) and extracellular matrix is shown in yellow arrows in **(E, F)**. Biofilm in the second condition of formation of *P. brasiliensis* (Pb18) and *M. tuberculosis* H37Rv (Pb18+H37Rv) in **(G–I)**. The blue arrows show the H37Rv bacilli and the orange arrows denote the presence of yeasts (PB18) in **(H, I)** and the presence of extracellular matrix is shown in yellow arrows in **(I)**.

SEM analysis revealed *M. tuberculosis* H37Rv (ATCC 27294) monospecies biofilms that exhibited an organized network of bacilli that connect to a highly extracellular polysaccharide matrix. In (**Figures 4A, B**), the yellow arrow shows the extracellular matrix (A) and the characteristics of the bacilli of the strain presented in the form of a network connected by the blue arrows in (B), and the 40Rv clinical strain presented a biofilm with a highly extracellular polysaccharide matrix that was more evident in the yellow arrows (C) with a consistent network of bacilli. However, some structural differences were observed such as a more compact structure and more irregular bacilli shapes and sizes when compared to the ATCC strain, as indicated by the blue arrows in (D). In the biofilm of *P. brasiliensis*, the yellow arrows show the high presence of extracellular polysaccharide matrix (E) involved in the entangled yeast, indicated by the orange arrows (F). In the analysis of mixed biofilms with two formation conditions, the first condition (H37Rv + Pb18) of mixed biofilm is shown in **Figures 5A–C**. A biofilm consistent with a network of bacilli and yeast, organized with a higher presence of extracellular matrix, is indicated by the yellow arrow (B) that surrounds the yeast Pb18, and the orange arrows (B and C) that connect to a complex network of H37Rv bacilli on the blue arrows in (B and C). The (40Rv + Pb18) mixed biofilm was formed in the first formation condition, as observed in (D–F), with a high presence of extracellular matrices involving the mixed biofilm microorganisms as indicated by the yellow arrows in (E and F), which involves a large amount of 40Rv bacilli as indicated by the blue arrows, and connecting the Pb18 yeasts as indicated by the orange arrows (E and F), however, this biofilm is more compact than the (H37Rv + Pb18) mixed biofilm. In the second condition of formation (Pb18 + H37Rv) mixed biofilm, it was observed in **Figures 5G–I** that the extracellular matrix involves the yeasts of Pb18, which is found at the base forming a carpet as shown in the orange arrows in (H and I) and on top of the yeasts. We observed that the H37 Rv bacilli had possible fragmentation in the blue arrows in H and I; this fragmentation is possibly due to the previous formation of the yeast.

### 3.3 Molecular Identification of *P. brasiliensis* and *M. tuberculosis* in Mixed Biofilm

**Figure 6:**
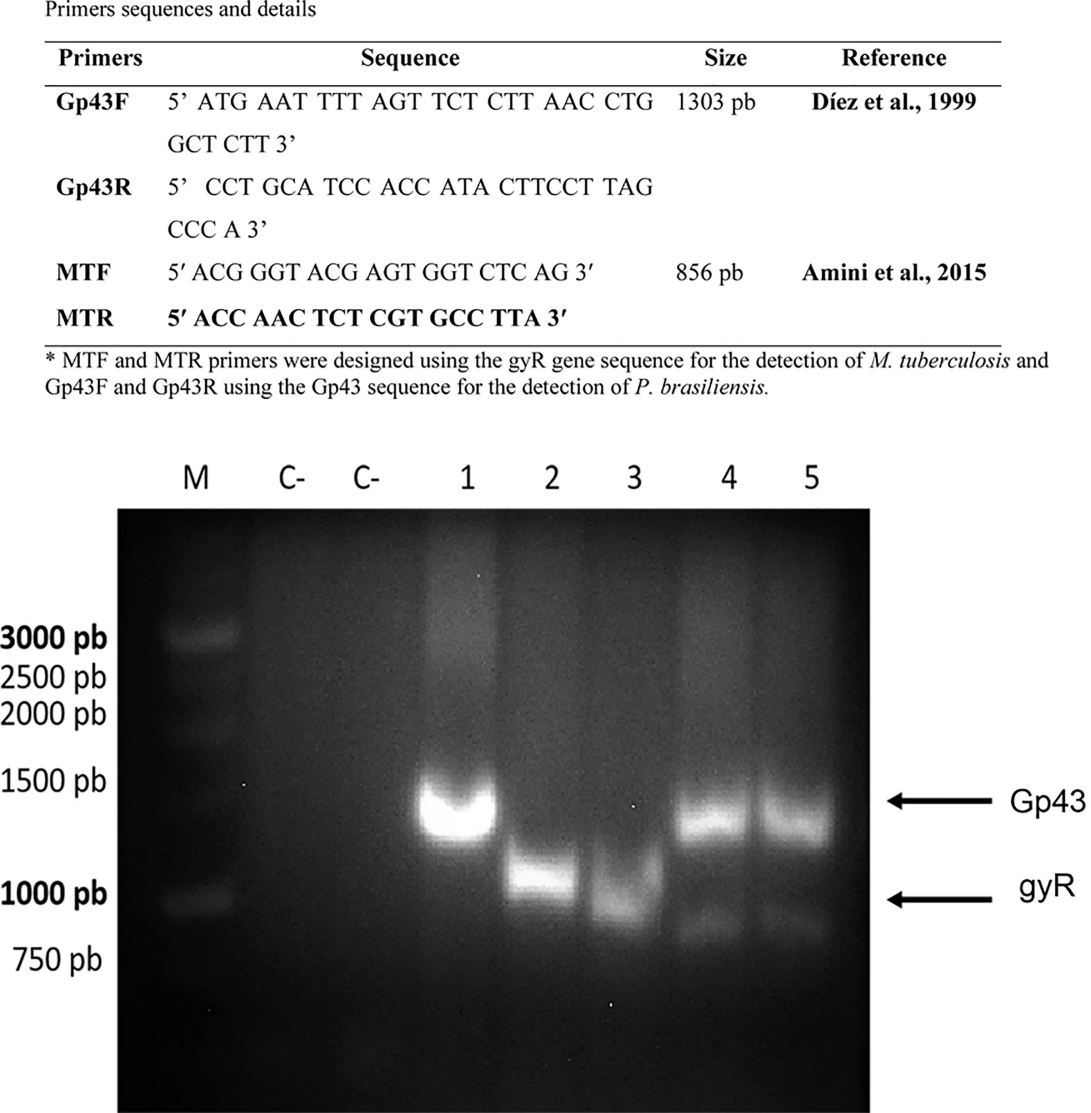
Multiplex PCR products on an electrophoresis gel. PCR with specific primers for *P. brasiliensis* and *M. tuberculosis.* M: marker (1 Kb DNA ladder, GeneDireX). C-: negative controls. Lane 1: *P. brasiliensis* (Pb18) – positive (1303 bp). Lane 2: *M. tuberculosis* ATCC (27294) – positive (856 pb). Lane 3: *M. tuberculosis* 40Rv– positive (856 pb). Lane 4: mix biofilm, *M. tuberculosis* ATCC (27294) and *P. brasiliensis* (Pb18) positives, Lane 5: *M. tuberculosis* 40Rv and *P. brasiliensis* (Pb18) positives mix biofilm, respectively.

PCR analysis of mixed biofilms: *M. tuberculosis* H37Rv (ATCC 27294) and *P. brasiliensis* (H37Rv + Pb18), and *M. tuberculosis* Rv40 clinical strain and *P. brasiliensis* (40Rv + Pb18). The PCR was used to identify *P. brasiliensis* and *Mycobacterium* strains respectively, as shown in **Figure 6** using the products of the PCR multiplex, as related to the amplification of both gp43 (*P. brasiliensis*) and gyR (*M. tuberculosis*) genes in the mixed biofilms, indicating the presence of both microorganisms.

### 3.4 Characterization of NE and NE3’chalc

#### 3.4.1 Mean Diameter, PDI, and Zeta Potential

**Figure 7:**
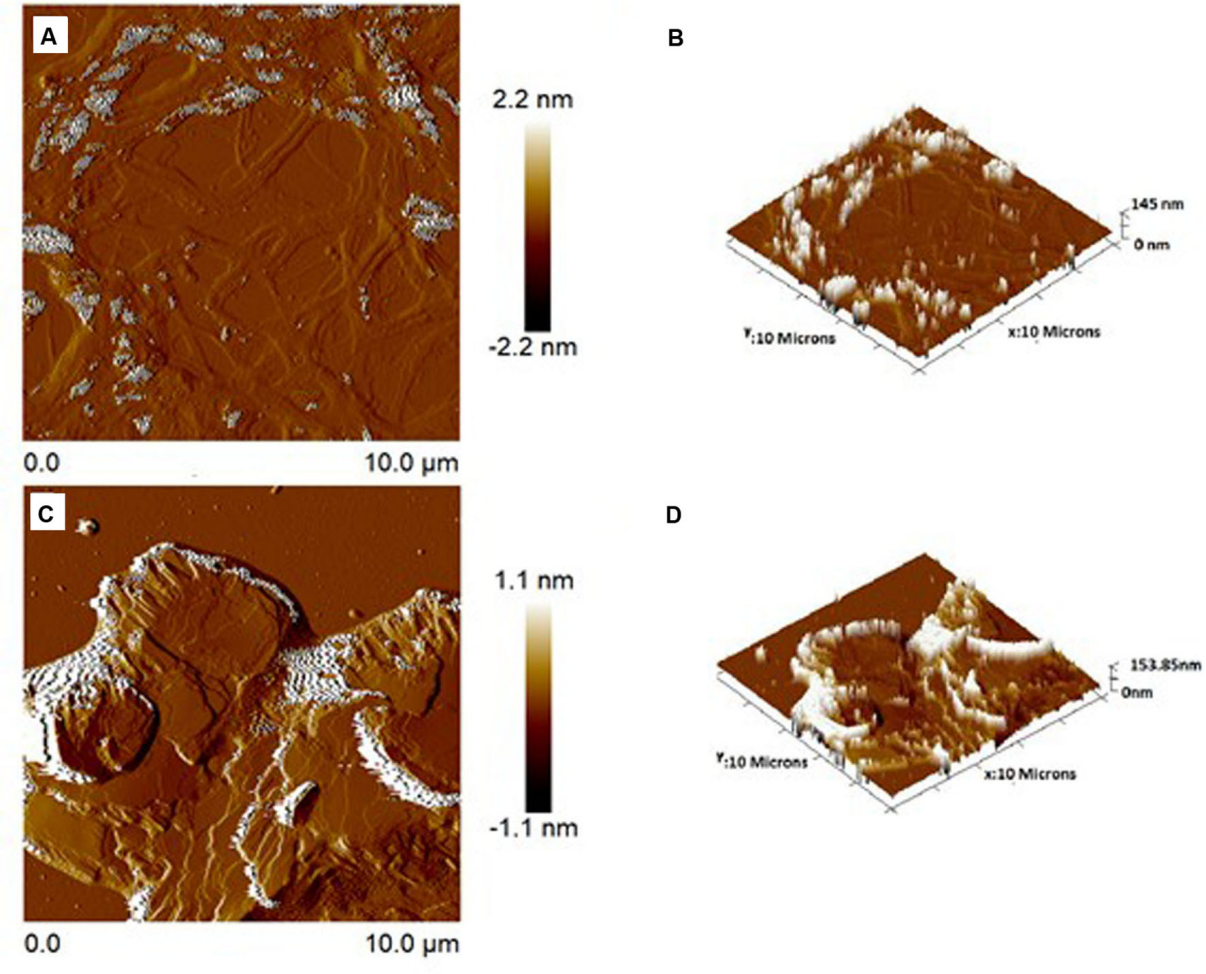
Atomic force microscopic images of surface topography 2-dimensional and 3-dimensional of NE **(A, B)**, respectively, and NE3’chalc **(C, D)**, respectively. The images were taken at room temperature.

**Figure 7A** shows that the 3'hidroxychalcone (3'chalc) was loaded into the NE to improve the solubility and stability of the drug, resulting in the formulation of NE3’chalc. The average diameter of NE drops was 145.00 ± 1.05 nm. The NE3’chalc produced had a size of 151.25 ± 0.60 nm. The polydispersity index (PDI) values were as follows: NE 0.20 ± 0.01 and NE3’chalc of 0.16 ± 0.01, showing homogeneity between the formulations in the particle sizes distributed in the measured samples. In addition to the zeta potential (ZP), the result for the control NLS was -58.20 ± 0.92 mV and for the formulation loaded with NE3’chalc was -56.10 ± 0.71 mV. All results showed no significant variation (*p < 0.05). The results are shown in **Table 1**.

**Table 1:** Characterization of the physicochemical properties of NE and NE3’chalc: particle size, polydispersity index (PDI), and zeta potential.

Formulations	Diameter ± SD (nm)	PDI ± SD	Potential Zeta ± SD
NE	145.00 ± 1.05	0.20± 0.01	-58.20 ± 0.92 mV
NE3’chalc	151.25 ± 0.60	0.16± 0.01	-56.10 ± 0.71 mV

NE, Nanoemulsion; NE3’chalc:3’hidroxychalcone loaded in nanoemulsion.

The value reported as mean ± SD; SD: Standard deviation, n = 10, the independent experiments.

#### 3.4.2 Atomic Force Microscopy

AFM was performed to assess the surface topography and size distribution images of the NE and 3´-hydroxychalcone (3’hydroxychalcone) loaded into the NE (NE3’chalc). The formulations were dispersed and the characteristic lamellas can be observed in both 2D and 3D topography images in both formulations, with an average particle size of 145 nm for NE (**Figures 7A, B**) and 153 nm for NE3’chalc (**Figures 7C, D**), corroborating previous results.

### 3.5 Cytotoxicity Assay

**Figure 8:**
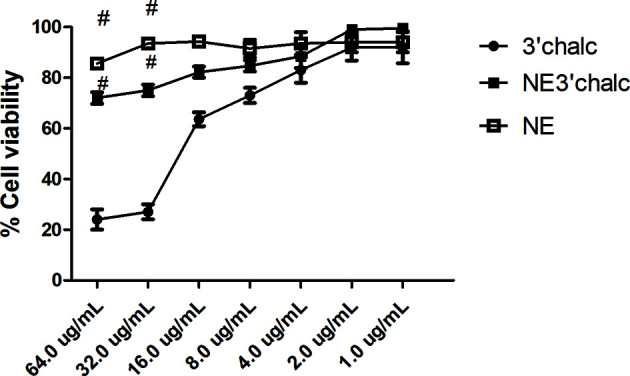
Percentage of cell viability of NE3’chalc, NE, and 3´chlac. The experiments were performed independently. There was a significant reduction in toxicity with NE3’chalc and 3'hidroxychalcone free, ^#^ = p < 0.001. NE, Nanoemulsion; NE3’chalc, 3'hydroxychalcone-loaded and 3’chalc, 3’ hydroxychalcone free.

The *in vitro* cytotoxicity assay was performed using the sulforhodamine B method to evaluate 3'chalc, NE3'chalc, and NE, for the HepG2 cell line. The 3'chalc showed cytotoxicity in this strain in a concentration-dependent manner, with 24% cell viability at 64.0 μg/mL, the highest concentration evaluated. In contrast, NE3'chalc achieved 72% cell viability at 64.0 μg/mL, reaching 100% viability at a concentration of 2.0 μg/mL. NE showed 86% cell viability at the highest concentration evaluated. The NE3'chalc and NE were not toxic and showed a significant difference (^#^p < 0.001) when compared with 3´chlac. These results demonstrate a decrease in cytotoxicity of the free drug 3’chalc when it was incorporated into NE, increasing cellular viability (**Figure 8**).

### 3.6 *In vitro* Antifungal and Antitubercularactivity of NE3’chalc

The susceptibility of *P. brasiliensis*, *M. tuberculosis* H37Rv (ATCC 27294), and 40Rv clinical strain against 3´ hidroxychalcone-free (3’chalc) and 3´hydroxychalcone loaded in NE (NE3’chalc) were evaluated for the minimum inhibitory concentration (MIC) values. The MIC values for 3’chalc free were 0.97 μg/mL for Pb 18 and 7.80 μg/mL for *M. tuberculosis* H37Rv (ATCC 27294) and 40Rv clinical strain, respectively. The NE 3’chalc improved the antifungal and antitubercular activity, with MIC values of 0.24 μg/mL for Pb 18 and 3.91 μg/mL for *M. tuberculosis* H37Rv (ATCC 27294) and 40Rv clinical strain, respectively. These values were 2–4 times higher than the MIC values of 3’chalc free. The NE showed no antifungal or antitubercular activity. Concerning the standard drugs, amphotericin B (AMB) showed MIC values of 0.13 μg/mL for (Pb 18) and > 62.50 μg/mL for *M. tuberculosis* H37Rv (ATCC 27294) and 40Rv clinical strain, demonstrating resistance against these microorganisms. The rifampicin (RIF) showed MIC values of 32.50μg/mL for (Pb 18), with low antifungal activity and with high activity against *M. tuberculosis* H37Rv (ATCC 27294) and 40Rv clinical strain, with an MIC value of 0.12μg/mL, respectively. The results are shown in **Table 2**.

**Table 2:** Antifungal and antitubercular activity (values expressed in μg/mL) 3'hydroxychalcone free and incorporated into the nanoemulsion (NE) and standard drugs.

Formulation	Pb18 MIC (μg/mL)	H37Rv (ATCC 2729)4) MIC (μg/mL)	Rv40 MIC (μg/mL)
3’hidroxychalcone	0.97	7.80	7.80
NE3’chalc	0.24	3.91	3.91
NE	62.50	>62.50	>62.50
AMB	0.13	>62.50	>62.50
RIF	32.50	0.12	0.12

MIC, Minimum inhibitory concentration; NE3’chalc, 3’hidroxychalcone loaded in nanoemulsion; NE, nanoemulsion; AMB, amphotericin B; RIF, rifampicin.

### 3.7 Effect of 3’chalc-Nanoemulsion Against Mature Monospecies and Mixed Biofilm

**Figure 9:**
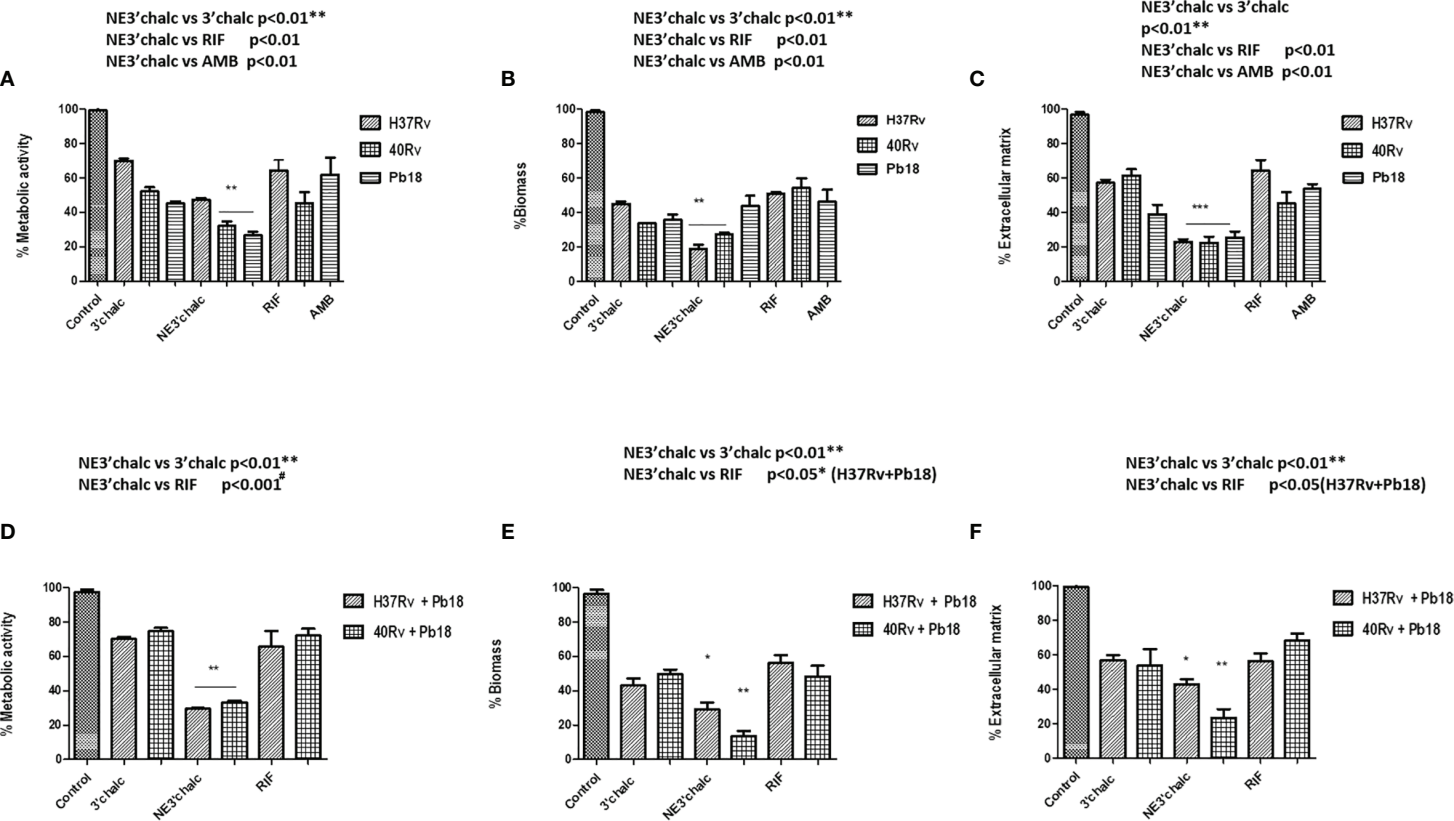
Anti-biofilm activity of 3’chalc and NE3’chalc against *M. tuberculosis* monospecies **(A–C)** and mixed biofilms (H37Rv + Pb18) and (40Rv + Pb18) **(D–F)**. The controls used were untreated (control), treatment control rifampicin (RIF) for monospecies and mixed biofilms and amphotericin B (AMB) for *P. brasiliensis* (Pb18) biofilm through the evaluation of the metabolic activity, quantification of biomass, and extracellular matrix, respectively. The experiments were performed independently, Mean ± SD was used in the data processing, and the differences between groups were analyzed for significance using ANOVA, followed by Tukey’s test for comparisons between groups, * = p < 0.05, ** = p < 0.01, *** = p < 0.001, were considered significant.

**Figure 10:**
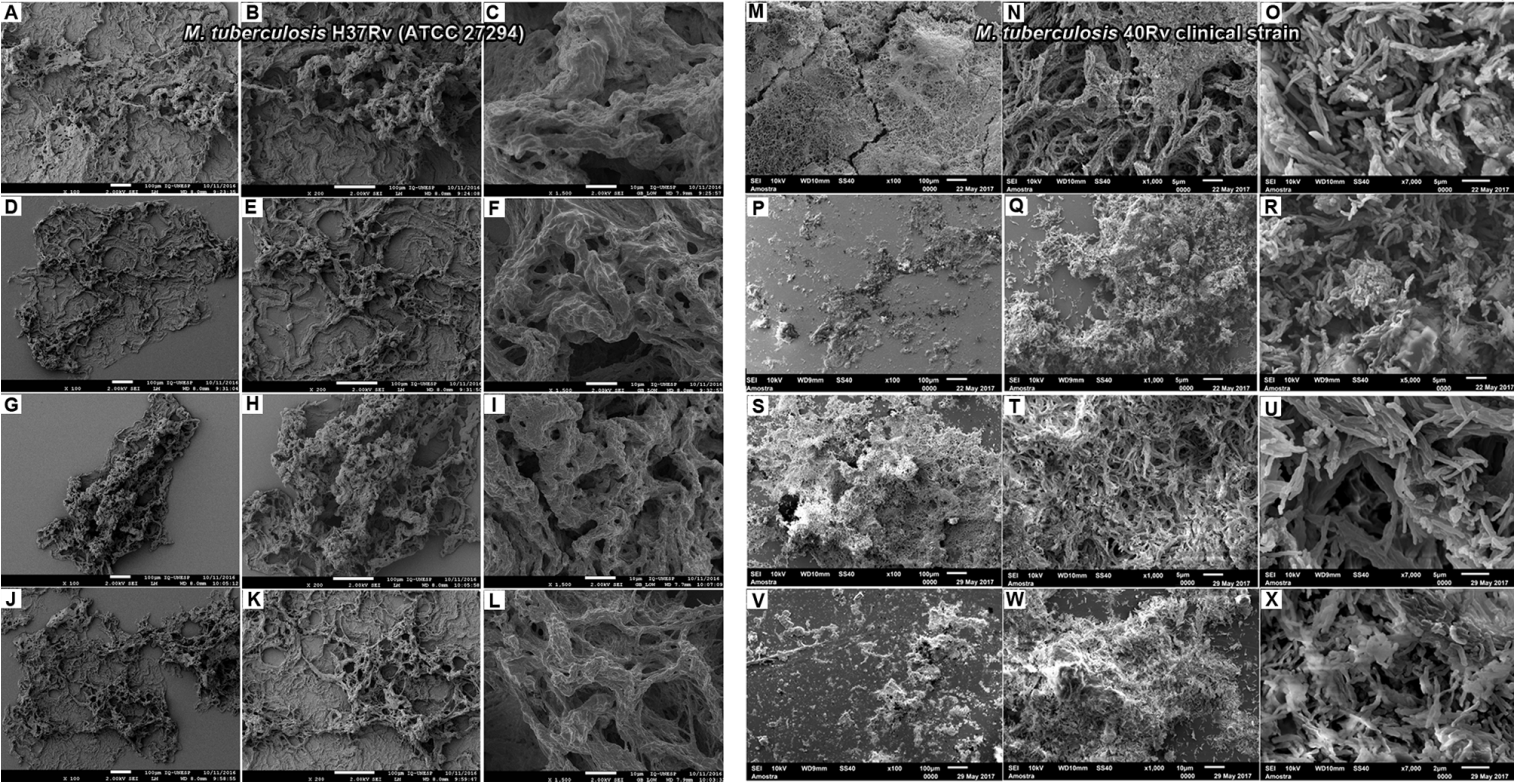
Electromicrographs of *M. tuberculosis* H37Rv (ATCC 27294) **(A–L)** biofilm. Untreated **(A–C)**, treatment with NE3'chalc **(D–F)**, 3’hidroxychalcone free **(G, I)** and rifampicin (RIF) as treatment control **(J–L)**; *M. tuberculosis* Rv40 clinical strain **(M–X)** Untreated **(M–O)**, treatment with NE3’chalc **(P–R)** and 3’hidroxychalcone free **(S–U)** and rifampicin **(V–X)** at a dose of 64µg/mL, respectively.

**Figure 11:**
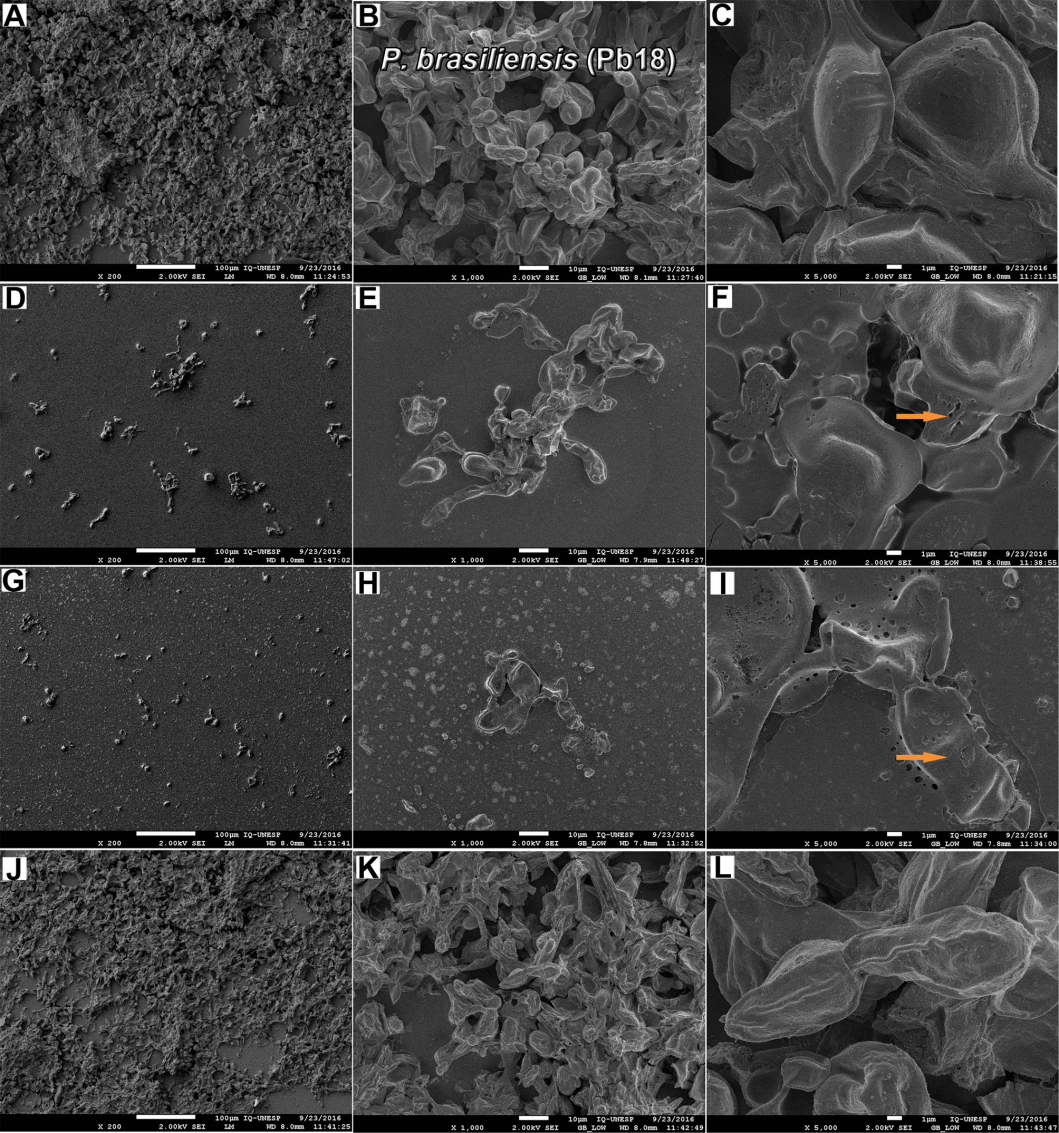
Electromicrographs of *P. brasiliensis* (Pb18) biofilm **(A–L)**. Untreated **(A–C)**, treatment with NE3’chalc at a dose of 15.6µg/m **(D–F)** and 3’hidroxychalcone free at a dose of 64µg/mL **(G–I)** and rifampicin (RIF) **(J–L)**. The orange arrows show some modifications structural in the biofilm.

**Figure 12:**
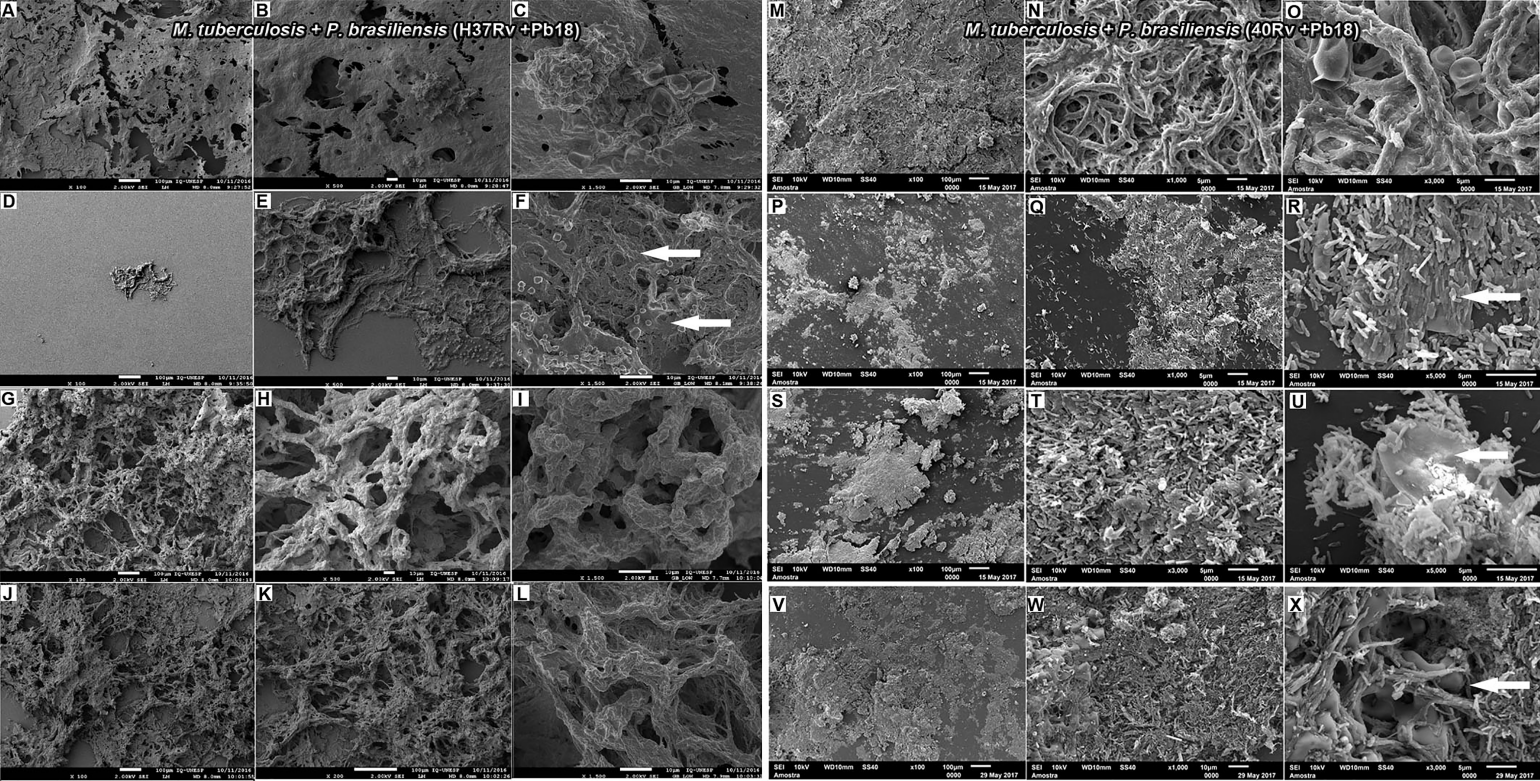
Electromicrographs of *M. tuberculosis* H37Rv (ATCC 27294 and *P. brasiliensis* (Pb18) (H37Rv+Pb18) mature **(A–L)** mixed biofilm. Untreated **(A–C)**, treatment with NE3’chalc **(D–F)** and 3’hidroxychalcone free **(D–F)** and and rifampicin (RIF) as treatment control **(J–L)**. *M. tuberculosis* Rv40 clinical strain and *P. brasiliensis* (Pb18) (40Rv+Pb18) **(M–X). (M–O)**, treatment with NE3’chalc **(P–R)** and 3’hidroxychalcone free **(S–U)** and rifampicin **(V–X)** at a dose of 64µg/mL, respectively. The blue arrows show some structural modifications in the biofilm when compared with the no treatment.

This evaluation was done using the XTT reduction assay to quantify the metabolic activities. The biomass and extracellular matrix were quantified using crystal violet and safranin stainings, respectively, on biofilms treated with 3'hidroxychalcone free (3'chalc) and 3'hidroxychalcone loaded in NE (NE3’chalc) against *P. brasiliensis*, *M. tuberculosis* H37Rv (ATCC 27294), and 40Rv clinical strain monospecies biofilms and their respective mixed biofilms (H37Rv + Pb18) and (40Rv + Pb18) formed in the first formation condition, because in the clinic we observed greater evidence of this association. The results are presented in **Table 3**. which shows the Sessile minimum 50% inhibitory concentration (SMIC_50_) was > 64.0–4.0 μg/mL fpr NE3’chalc and > 64.0–32.0 μg/mL for 3´hidroxychalcone free. rifampicin was used as a treatment control for *M. tuberculosis* biofilms; however, it was not used for the *P. brasiliensis* monospecies biofilm, since this drug did not present antifungal activity against the strain that was evaluated. Thus, amphotericin B (AMB) was used as a control for this strain. The result show an RIF of > 64.0 μg/mL and AMB of > 64.0–64.0 μg/mL. The NE3’chalc achieved a maximum reduction of 8 times more than the metabolic activity and production of extracellular matrix and 4 times more than the biomass SMIC_50_ when compared with the drug-free. The NE3’chalc shows better antibiofilm activity than the free drug (3’chalc free) and the controls used in the treatments were RIF and AMB. In the treatment of all biofilms, they were evaluated through the quantification of metabolic activity, quantification of biomass, and extracellular matrix. The treatment of the monospecies biofilms of *M. tuberculosis* and *P. brasiliensis*, previously reported with (NE3’chalc) showed the best results in the three measurements, with 27%, 19%, and 14% for metabolic activity, biomass, and extracellular matrix, respectively, which represents a reduction of 73%, 81%, and 86%, respectively. Comparing the NE3’chalc with 3’chalc free, RIF, and AMB, the results show a significant difference (^**^ = p < 0.01) in the previously reported quantifications (**Figures 9A–C**). Similarly, NE3'chalc showed the best-mixed antibiofilm activity, with the results of metabolic activity, biomass quantification, and extracellular matrix being 29.29% and 57% for the mixed biofilm H37Rv + Pb18 (representing a reduction of 71% and 43% , respectively) and 33%, 14%, and 24%, respectively, representing reductions of (67%, 86%, and 76%, respectively), for the 40 Rv and Pb18 mixed biofilms. Comparing the NE3’chalc and 3’chalc results, the significant difference was ^**^ = p < 0.01. When NE3’chalc was compared with RIF, the significant difference was ^#^ = p < 0.001 in the quantification of metabolic activity in the evaluation of 40Rv + Pb18. The quantification of biomass and extracellular matrix showed a significant difference of ^*^ = p < 0.05 in the evaluation of the biofilm H37Rv + Pb18. These results show that NE3’chlac had a better antibiofilm activity, as shown in (**Figures 9D–F**). To confirm the activities of NE3'chalc and 3'chlac free, SEM was used to check the possible damage to the biofilm through topographical analysis. We evaluated 3’chalc free, NE3’chalc, and RIF or AMB as the treatment controls depending on the biofilm formed (**Figures 10**–**12**). All treatments carried out against monospecies and mixed biofilms were carried out at a concentration of 64 μg/mL, except for the evaluation of NE3’chalc against *P. brasiliensis* which was carried out at a concentration of 16 μg/mL (**Figures 11D–F**). In the evaluation of *M. tuberculosis* H37Rv biofilm, as shown in (**Figures 10A–L**), no treatment was used as a control in (A-C), with NE3’chalc treatment in (D-F), 3'chalc treatment in (G-I), and RIF as treatment control in (J-L). It was observed that NE3’chalc showed the greatest reduction in biofilm with the rupture of membranes and leakage of cellular components of the biofilm when compared to the control with no treatment, the free drug 3’chalc free, and the RIF treatment control. For the evaluation of treatment of *M. tuberculosis* 40Rv biofilm in (**Figures 10 M–X**), no treatment was used as a control in (M-O), with NE3’chalc treatment in (P-R), 3'chalc free treatment in (S-U), and RIF as treatment control in (V-X). It was observed that NE3’chalc almost completely eliminated the biofilm, with membrane rupture and an almost total reduction in the biofilm biomass. Compared to 3’chalc free and RIF, a greater reduction in the biofilm, mainly with the free drug, was observed. We evaluated the treatment of *P. brasiliensis* biofilm as shown in (**Figures 11 A–L**), no treatment in (A-C), with NE3’chalc treatment in (D-F) and 3´chalc free treatment in (G-I), and AMB as treatment control in (J-L). The results showed that NE3’chalc caused almost total elimination at a concentration of 16 μg/mL, and this was only observed at the concentration of 64 μg/mL for 3’chalc free, with a marked structural modification in the membrane of yeasts compared to the untreated control, mainly with 3’chalc free, as shown with the orange arrows in (F) for NE3’chalc and (I) for 3´chalc free. In addition, NE3’chalc showed the greatest reduction in biofilm as compared to the AMB treatment control. The results showed that NE3'chalc showed better antibiofilm activity than the 3'chalc free and RIF and AMB treatment controls used in their respective biofilms, thus corroborating the quantification results obtained in the treatment of monospecies biofilms to evaluate the treatment of the mixed biofilms (H37Rv + Pb18) and (40Rv + Pb18). Topographic analysis of these biofilms was also performed. Comparisons were made for the following treatments of mixed biofilm (H37Rv + Pb18) (**Figures 12A–L**): no treatment (A-C), NE3’chalc treatment (D-F), 3'chalc free treatment (G-I), and RIF as treatment control (J-L). NE3’chalc produced the near elimination of the mixed biofilm of H37Rv + Pb18, with structural modifications, extravasation of cell content, and a large reduction in biomass observed. In addition, a structural modification in the bacilli and no presence of yeast was observed when compared to the untreated control indicated with the white arrows in (F); the apparent absence of yeast *P. brasiliensis* was observed in the mixed biofilm. The mixed biofilm 40Rv + Pb18 (**Figures 12M–X**) was tested with untreated control (M-O), NE3'chalc treatment (P-R), 3'chalc free treatment (S-U), and RIF as treatment control (V-X). Similar to the previous evaluation, NE3'chalc showed an almost complete reduction in the biofilm. In addition, it was observed that the bacilli do not have the same morphological characteristics as the control without treatment as indicated by the white arrow (R), and we observed no presence of yeast compared to the no treatment control (M-O). It was also observed that the 3’chalc free treatment showed a smaller reduction in biofilm biomass than the NE3’chalc, with a large reduction in bacilli and yeasts, as indicated by a white arrow (U). The RIF control showed a reduction in biofilm comparable to 3’chalc; with a modification to the yeast, they were completely shapeless (X). The topographic analysis corroborated the previous results and the NE3’chalc showed the best antibiofilm activity against the monospecies and mixed biofilms.

**Table 3:** Sessile minimum inhibitory concentration values, expressed in μg/mL, of 3'hydroxychalcone free (3'chalc) and 3'hidroxychalcone loaded into nanoemulsion (NE3’chalc) capable of reducing at least 50% of the metabolic activities, biomass, and matrix extracellular of mature monospecies of *P. brasiliensis* (Pb18), *M. tuberculosis* (H37Rv ATCC 27294), and 40Rv clinical strains and mixed biofilms, (H37Rv + Pb18) and (40Rv +Pb18) biofilms.

	*P. brasiliensis* (Pb18)	H37Rv (ATCC27294)	40Rv	H37Rv +Pb18	40Rv +Pb18
XTT (SMIC_50_)					
3’hidroxychalcone	64.0	>64.0	>64.0	>64.0	>64.0
NE3’chalc	8.0	64.0	16.0	32.0	32.0
RIF	>64.0	>64.0	64.0	>64.0	>64.0
AMB	>64.0	–	–	–	–
Crystal Violet (SMIC_50_)					
3’hidroxychalcone	64.0	>64.0	>64.0	64.0	64.0
NE3’chalc	64.0	16.0	16.0	16.0	16.0
RIF	>64.0	>64.0	>64.0	>64.0	>64.0
AMB	64.0	–	–	–	–
Safranin (SMIC_50_)					
3’hidroxychalcone	32.0	>64.0	>64.0	64.0	64.0
NE3’chalc	8.0	16.0	8.0	16.0	8.0
RIF	>64.0	>64.0	>64.0	>64.0	64.0
AMB	64.0	–	–	–	–

SMIC_50_ (sessile minimum inhibitory concentration capable of reducing at least 50% of mature biofilms).

## 4 Discussion

*M. tuberculosis* biofilms play an important role in the process of caseous necrosis and cavitation formation in the lung tissue (Kulka et al., 2012b; Basaraba and Ojha, 2017). In addition, *M. tuberculosis* can coexist with other mycobacteria, bacteria, and fungi, and its ability to form biofilms probably gives it the ability to communicate with other microorganisms to form complex biofilms (Flores-Valdez, 2016; Brennan, 2017). It can coat medical devices in hospitalized and immunologically compromised patients, enabling the formation of coinfection. *P. brasiliensis* is increasingly recognized by clinicians as comorbidities can occur simultaneously in up to 20% of patients (Quagliato et al., 2007; Shikanai-Yasuda et al., 2017). These interactions may be the key to understanding the development of new treatments (Flores-Valdez, 2016). Thus, we evaluated the formation and treatment of mixed biofilms of *M. tuberculosis* and *P. brasiliensis*. Colorimetric assays were initially performed to evaluate the metabolic activity, quantification of biomass and extracellular matrix, and essential parameters for biofilm formation, which is an important tool for assessing the viability of eukaryotic and prokaryotic cells (Costa-Orlandi et al., 2014). The *P. brasiliensis* monospecies biofilm showed high metabolic activity, biomass, and extracellular matrix within 144 h of formation **(** **Figures 2A, B** **)**. These results are corroborated by previous studies (de Cássia Orlandi Sardi et al., 2015; Cattana et al., 2017; Oliveira et al., 2020). The evaluation of metabolic activity, biomass, and extracellular matrix of *M. tuberculosis* H37Rv (ATCC) and the 40 Rv clinical strain presented a strong and mature biofilm within 45 days of formation, showing a high survival and leading 45 days to be recognized as the optimal amount of time to allow for formation. This evaluation also showed that it did not present a significant difference (* = p < 0.05) in the three evaluations carried out when compared with ATCC and the clinical strain, showing a similar pattern of behavior between all three **(** **Figures 2C** and **3A, C** **)**. Previous studies have demonstrated the formation of a biofilm of *M. tuberculosis* at 5 weeks and observed a strong propensity to grow in organized multicellular structures, with biomass production being directly related to biofilm survival (Kulka et al., 2012; Trivedi et al., 2016). The mixed biofilms formed in the first association formed with *M. tuberculosis* H37Rv (ATCC27294) and 40Rv (clinical strain) were initially incubated following the subsequent assistance of *P.brasiliensis* forming the (H37Rv + Pb18) and (40Rv + Pb18) biofilms (**Figure 1B.1**). This association was considered in cases of comorbidity reported in patients who had TB and with compromised immune systems who acquired PCM, which is an opportunistic disease (Torres-Pereira et al., 2009; Martinez, 2017). These mixed biofilms also showed high metabolic activity, biomass production, and extracellular matrices. In general, the curves increased constantly from day 20 after formation until day 45 at the complete formation of the mature mixed biofilm. The results of these evaluations were comparable to those of the monospecies biofilms and showed no significant difference (^*^ = p <0.05) in metabolic activity assessment and biomass quantification. However, the extracellular matrix did show a significant variation between mixed biofilms and H37+Pb18 and 40Rv monospecies biofilms as shown in **Figures 2C**, **3A, C**, implying that the microorganisms present in the mixed biofilms did not interfere completely with the development of each other in this interaction. Thus, the multispecies biofilms presented different behaviors and m benefit from co-aggregation, which enables them to compete, or have antagonistic interactions (Rodrigues et al., 2020). In the development of polymicrobial biofilms, consisting of fungi and bacteria, *C. albicans* can develop together with *Staphylococcus aureus* and *Staphylococcus epidermidis* can also develop together with *Enterococcus* spp. in the course of blood-borne infections (Harriott and Noverr, 2010; Kim et al., 2012).

The second condition of biofilm (PB18 + H37Rv) formation consists of the initial formation of Pb18 with the subsequent addition of *M. tuberculosis* (**Figure 1B.2** **).** This association was due to the initial comorbidity cases of patients with PCM who acquired tuberculosis due to their critical condition (Nunura et al., 2010; Guery et al., 2015). The results showed a high quantification of metabolic activity, biomass, and extracellular matrix during the 30 days of formation, with a decay in production occurring within 35 days that continued until the last day of formation at 45 days. This association showed no significant difference ^*^ = p < 0.05 when compared to their monospecies biofilms in the three evaluations; however, the metabolic activity showed a significant difference in a single time within 40 days of formation (^**^ = p < 0.01). This association did not significantly interfere with the development of one or both microorganisms present in the mixed biofilm in (**Figures 2D**, **3B, D**). Therefore, the order of formation of microorganisms in a biofilm is an important factor of formation, whether it is bacteria or fungi. The production of biomass and the extracellular matrix can change, with greater or lesser production of these components by each species individually (Foster and Bell, 2012; Ren et al., 2015; Tan et al., 2017; Wijesinghe et al., 2019). A study conducted by Rodrıguez-Sevilla et al. (2018) demonstrated the formation of monospecies and dual-species of *M. abscessus* and *P. aeruginosa*. This evaluation showed a difference between the monospecies mycobacterial biofilm and its dual-species after 24 h of formation, slightly modifying the formation (Rodríguez-Sevilla et al., 2018).

Following the SEM analysis, it was shown that clinical biofilm strains of *M. tuberculosis* H37Rv in (**Figures 4A, B**) and 40Rv in (**Figure 4C, D** **)** formed biofilms on the polystyrene surface for 45 days in the presence of high biomass, with a consistent and dense network of characteristic bacilli growing in all strain directions and covered by an extracellular polysaccharide (EPS) matrix that constitutes the ultrastructure of a mature biofilm. *M. tuberculosis* 40Rv proved to be a more compact biofilm than H37Rv and showed a greater amount of EPS, especially in some points where greater accumulation was observed. The 40Rv bacilli showed different sizes and dimensions, unlike the H37Rv bacilli which were more homogeneous. Biofilms formed by mycobacteria have the same definition as other biofilms and can develop these structures on surfaces as well as at aerial interfaces (Ojha et al., 2008). Canetti discovered a biofilm of *M. tuberculosis in vivo* which exhibited dense layers of bacilli that did not adhere to the surface within the granuloma (Canetti, 1965; Bacon et al., 2014). Among the factors involved in the formation of *M. tuberculosis* biofilm, PE11 is a lipase associated with the cell wall expressed by Mtb both *in vitro* and *in vivo*, modulating its lipid profile and cell wall architecture, such as adding mycolic acids and threolase esters (Islam et al., 2012; Rastogi et al., 2017). In addition, the monospecies *P. brasiliensis* biofilm in (**Figures 4E, F** **)** showed a solid network of yeast embedded with an extracellular polysaccharide matrix (EPS), which has been shown by our research group, indicating that *P. brasiliensis* can form a biofilm (de Cássia Orlandi Sardi et al., 2015). The biofilm of *Paracoccidioides* spp. was demonstrated *in vivo* in a 63-year-old patient in the aorto-bifemural vascular prosthesis after implantation in the patient, as confirmed using SEM (Cattana et al., 2017; Lorente-Leal et al., 2019).

The mixed biofilms (H37Rv + Pb18) in (**Figures 5A–C**) and (40Rv + Pb18) in (**Figures 5D–F** **)** presented a dense structure where the *M. tuberculosis* bacilli unit formed a highly organized network of bacilli that connect with the yeasts, creating a coexistence between the two microorganisms. However, the mixed biofilm formed with the clinical strain (40Rv + Pb18) revealed a more compact biofilm formation, with a greater accumulation of EPS at some points. This appeared in larger blocks, and the yeasts were more intertwined than in the mixed biofilm (H37Rv + Pb18). According to Costa et al. (2020) the study was carried out in hemodialysis samples from hospital environments; biofilms of single and double species of *Candida parapsilosis* and *M. smegmatis* were found where this bacterium benefits from its association with *C. parapsilosis*, which retards the growth of the fungus and allows the coexistence of microorganisms (Costa et al., 2020). Another study demonstrated the interaction of *C. albicans* with *Streptococcus mutans* microorganisms present in periodontitis lesions, which showed synergism with an increase in the virulence of biofilm (Falsetta et al., 2014; Chevalier et al., 2018).

In contrast, in the second combination of mixed biofilm (Pb18 + H37Rv) in (**Figures 5G–I**), a large number of open yeasts of *P. brasiliensis* was observed at the base of the biofilm, connecting to the fragmented bacilli and forming a compact and complex structure; thus, characterizing the mature biofilm. Although the quantification results did not show a great interference in biofilm formation, in this evaluation, this interference becomes more evident. This is due to the previous formation of the biofilm, *P. brasileinsis*, which can produce adhesins, such as GP43 and 30 kDa, and virulence proteins, such as glyceraldehyde-3-phosphate-dehydrogenase (GAPDH), involved in the pathogenesis of the fungus, and are important factors for its formation (de Cássia Orlandi Sardi et al., 2015). Other studies have shown that *C. albicans and P. aeruginosa* exhibit competitive action and antagonistic behavior; this interaction has a distinct clinical implication if they occur in an immunocompromised host (Shirtliff et al., 2009; Bernard et al., 2019).

In agreement with previous results, the order of formation is an important parameter for determining the coexistence or prevalence of one of the microorganisms present in the mixed biofilm. Polymerase chain reaction (PCR) was performed to identify the microorganisms present in the mixed biofilm in all associations. According to our results, multiplex PCR confirmed the presence of *M. tuberculosis* H37Rv (ATCC 27294), 40Rv (clinical strains), and *P. brasiliensis*, which are involved in the formation of mixed biofilms (**Figure 6**). According to Díez et al. (1999), PCR specifically targets the gp43 protein that is widely used in the identification and diagnosis of adhesion and biofilm formation in *P. brasiliensis* (Correa et al., 2007; Reis et al., 2008; de Cássia Orlandi Sardi et al., 2015).

Amini et al. (2015) evaluated samples from 100 patients using bronchoalveolar lavage samples from patients with suspected TB using multiplex PCR, with a specific gyR gene target used in the identification of *M. tuberculosis*. They obtained a specificity of up to 80 and a sensitivity of 97.8% (Amini et al., 2015; Sharma et al., 2018), confirming the presence of both microorganisms studied in the mixed biofilm.

Owing to the lack of treatment for this coinfection and the toxic effects of the standard drugs used in the treatment of TB and PCM, there is a need for new treatment alternatives to reduce side effects and increase their biological activity (Brime et al., 2004; Ferrara et al., 2011; Combrink et al., 2020). NE are controlled drug release systems that allow greater bioavailability and stability of the administered drug (Souza et al., 2015; Medina-Alarcón et al., 2017b). The main reason why 3’chalc was incorporated into NE was to improve its physicochemical characteristics and increase its antibiofilm activity (Medina-Alarcón et al., 2020).

The results of the present study showed that the formulations evaluated were thermodynamically stable and translucent, and all are within the parameters of normality observed in **Table 1**. The nanoemulsions (NE) had a particle size of 145.0 nm and NE3’chalc (nanoemulsion loaded with 3’hydroxichalone) had a diameter of 151.25 nm. Both formulations were within the range of 10-200 nm, hence the formulations studied are within the defined value (Goyal et al., 2012; Medina-Alarcón et al., 2017b; Haider et al., 2020). Another parameter of physicochemical characterization is the polydispersity index (PDI), which is calculated by dividing the average droplet size by the average number of droplets measured for each formulation. The value obtained for all formulations was 0.20 for (NE) and 0.16 for NE3’chalc. Several studies have reported that the ideal distribution of the (PDI) is the value (< 0.5); therefore, these results reflect the homogeneity in the formulations (da Silva et al., 2014). The potential zeta of the formulations under study are within the parameters of normality: the values were -58.20 and -56.10 mV for NE and NE3’chalc respectively, and the reference values range from -30–30 mV. This parameter allows forecasts on the stability of colloidal dispersion storage (Wang et al., 2006; Araújo et al., 2011). Another physicochemical characterization parameter was morphological and distribution evaluation through AFM, a widely used technique for nanostructured lipid system (SNL) evaluation (Starostina et al., 2008). The evaluated formulations of NE and NE3’chalc showed homogeneous systems with lamellar characteristics, and particle size distribution for NE within 145 nm and NE3’chalc within 153.85 nm (**Figures 7A–D** **)**, which is consistent with previous results, showing formulations within the normality parameters in the evaluation of NE and nanoparticles (Marquele-Oliveira et al., 2016).

In order to reduce the cytotoxicity and increase its antimicrobial activity, the cytotoxicity assay was performed in which our results showed that NE and NE3’chalc have no toxic effect and NE3’chalc reduces the cytotoxicity of the free drug, increasing its cellular viability by 48%. These results (**Figure 8** **)** show that the NE are biocompatible formulations and that the lipids used in the formulation are well tolerated by eukaryotic cells (Medina-Alarcón et al., 2020). In addition to the reduction in cell uptake, their toxic effects were reduced by reducing the dose to the host (Nakamura et al., 2018).

Subsequently, the antifungal and antitubercular activity of NE3’chalc presented MIC values at concentrations lower than that of 3’chalc, thus presenting a better antifungal and antitubercular action than the free drug and showed a better antifungal action than the control RIF used in the treatment. The NE was also evaluated, and it did not show antifungal or antitubercular activity, which demonstrates that it does not interfere with the action of 3’chalc (**Table 2** **)**. This was due to the efficient increase in the release of drugs when incorporated into the NE, thereby increasing drug solubility, biological activity, and bioavailability (Kawakami et al., 2002; Thakur et al., 2013; Hertiani et al., 2019). This result can be facilitated by the lipid composition of NE, such as cholesterol and phosphatidylcholine, which easily interact with the fungal or bacterial membrane, thus destabilizing their integrity and allowing an increase in drug permeability and the consequent death of microorganisms (Sha et al., 2005; Nirmala et al., 2013; Nikonenko et al., 2014; do Carmo Silva et al., 2020). Medina-Alarcón et al. (2020) demonstrated an increase in the antifungal activity of 2’hydroxychalcone when incorporated into a NE, managing to significantly increase its activity against *Paracoccidioides* spp. (Medina-Alarcón et al., 2020).

Owing to the antibacterial and antifungal activity presented by NE3’chalc, the antibiofilm activity of this formulation was evaluated against the monospecies biofilms of *P. brasiliensis* and *M. tuberculosis* (ATCC H37Rv and Rv40 clinical strain) and the mixed biofilms formed in the first condition of association (H37Rv + Pb18 and 40Rv + Pb18). According to our results, NE3’chalc showed the best results in evaluating the reduction in metabolic activity, amount of biomass, and extracellular matrix of monospecies and mixed biofilms. Their SMIC_50_ values showed a reduction in their concentrations by 4–8 fold for monospecies biofilms and 2-8 fold for mixed biofilms when compared to free 3’chalc (**Table 3** **)**. In addition, NE3’chalc showed a greater reduction in metabolic activity, biomass, and extracellular matrix significantly (^#^p < 0.001), managing to reduce the metabolic activity of the mixed biofilm up to 42% more when compared to 3’chalc free and with the RIF treatment control (**Figure 9**). The high lipophilicity of the NE components allows it to effectively interact with the biofilm cell membrane, which is highly lipophilic and allows for a change in cell permeability and consequent death of the microorganisms (Andrade-Ochoa et al., 2015).

Similarly, Rossi et al. (2017) evaluated *Cymbopogon flexuosus* NE against *M. fortuitum* (ATCC 6841), *M. massiliense* (ATCC 48898), and *M. abscessus* (ATCC 19977) in planktonic and biofilm form, showing antibiofilm action mainly in its nanostructured form (Rossi et al., 2017).

Finally, the SEM evaluation results for NE3’chalc corroborate our previous results and show that NE3’chalc obtained the best results when compared to 3’chalc and RIF, with the near elimination of the biofilm with biofilm rupture, leakage of components cells, and almost complete elimination of monospecies and mixed biofilm, and with some structural alterations of the yeast membrane in the*P. brasiliensis* biofilm and the mixed biofilm (**Figures 10** **–** **12**).

Because the systems can act as drug reservoirs, which allow the release of the active principle from the internal to the external phases, the process of adhesion of the microorganism is possible due to the oil phase of the system and is involved in the interaction with the cell membrane components of the microorganism, such as ergosterol, mycolic acids, and other membrane lipids, allowing the destabilization of the integrity and consequent lysis and death of the microorganism in planktonic form or biofilm (Aiemsaard et al., 2011; Nirmala et al., 2013; Rossi et al., 2017). In addition, Song et al. (2016) showed that MRSA cells were analyzed using microscopy scanning electronics, where bacteria treated with CHX (nanoemulsion) at a concentration of 0.5 µg/mL showed homogeneously distributed cytoplasm after treatment with NE, and a change was observed in the cell that led to a change in the permeability of the cell membrane (Song et al., 2016). NE3’chalc had the highest antibiofilm activity and demonstrated that the system is extremely promising for the treatment of monospecies biofilms and especially mixed biofilms.

Given the above, we can conclude that our results are extremely important, as they show for the first time the formation of mixed biofilms of *M. tuberculosis* and *P. brasiliensis*, which are microorganisms responsible for serious pulmonary infections that can lead to the death of patients due to lack of adequate treatment. In addition, our research highlights NE3’chalc, which has been shown to be an effective alternative, increasing its solubility, stability, and antibiofilm activity, and may become a promising new alternative for the treatment of mixed biofilms of these microorganisms. This is of great relevance, since there is a lack of treatment for these microorganisms that present themselves as comorbidities, especially in immunocompromised patients. More trials will be needed in the future to complement our research.

## Data Availability Statement

The raw data supporting the conclusions of this article will be made available by the authors, without undue reservation.

## Author Contributions

KM-A performed the tests: monospecies and mixed biofilm formation assay, characterization of all biofilms, preparation of NLS, characterization of the physicochemical properties of the nanoemulsion, evaluation of antifungal, antibacterial, and antibiofilm activity, and writing of the manuscript. IT and GF evaluated the antifungal and antibiofilm activity of *P. brasiliensis*. MP-d-S performed the AFM analysis. CM performed the PCR analysis. MS prepared and characterized the 3’hydroxychalcone. LR professor guided in the preparation and characterization of the 3’hydroxychalcone. MC professor of nanotechnology oriented in the preparation and characterization of the nanoemulsion and corrected the manuscript. FP professor guided in mycobacterial assays. MM-G professor guided in the biological tests and corrected the manuscript. AF-A professor guided in the biological assays and corrected the manuscript. All authors contributed to the article and approved the submitted version.

## Funding

This research was funded by the Coordenacão de Aperfeiçoamento Pessoal de Nıvel Superior (CAPES). Pró-Reitoria de Pós-Graduação (PROPG), PDI-UNESP, Conselho Nacional de Pesquisa e Desenvolvimento (CNPq), Programa de Apoio ao Desenvolvimento Científico e Tecnologico (PADC- UNESP), Fundaçao de Amparo a Pesquisa do Estado de São Paulo (FAPESP; grant number: 2013/05853-1).

## Conflict of Interest

The authors declare that the research was conducted in the absence of any commercial or financial relationships that could be construed as a potential conflict of interest.

## Publisher’s Note

All claims expressed in this article are solely those of the authors and do not necessarily represent those of their affiliated organizations, or those of the publisher, the editors and the reviewers. Any product that may be evaluated in this article, or claim that may be made by its manufacturer, is not guaranteed or endorsed by the publisher.
